# GTMNet: a vision transformer with guided transmission map for single remote sensing image dehazing

**DOI:** 10.1038/s41598-023-36149-6

**Published:** 2023-06-07

**Authors:** Haiqin Li, Yaping Zhang, Jiatao Liu, Yuanjie Ma

**Affiliations:** School of Information Science and Technology, Yunnan Normal University, Kunming, 650500 Yunnan China

**Keywords:** Ecology, Ecology, Natural hazards

## Abstract

Existing dehazing algorithms are not effective for remote sensing images (RSIs) with dense haze, and dehazed results are prone to over-enhancement, color distortion, and artifacts. To tackle these problems, we propose a model GTMNet based on convolutional neural networks (CNNs) and vision transformers (ViTs), combined with dark channel prior (DCP) to achieve good performance. Specifically, a spatial feature transform (SFT) layer is first used to smoothly introduce the guided transmission map (GTM) into the model, improving the ability of the network to estimate haze thickness. A strengthen-operate-subtract (SOS) boosted module is then added to refine the local features of the restored image. The framework of GTMNet is determined by adjusting the input of the SOS boosted module and the position of the SFT layer. On SateHaze1k dataset, we compare GTMNet with several classical dehazing algorithms. The results show that on sub-datasets of Moderate Fog and Thick Fog, the PSNR and SSIM of GTMNet-B are comparable to that of the state-of-the-art model Dehazeformer-L, with only 0.1 times of parameter quantity. In addition, our method is intuitively effective in improving the clarity and the details of dehazed images, which proves the usefulness and significance of using the prior GTM and the SOS boosted module in a single RSI dehazing.

## Introduction

Remote sensing satellites and unmanned aerial vehicle (UAV) sensors are susceptible to atmospheric phenomena that can impair the contrast and color fidelity of the collected images, resulting in weakened image details and making it difficult to recognize information in the image. Haze, fog and smoke are very common atmospheric phenomena generated by atmospheric absorption and scattering. With the application of remote sensing technology in the fields of police security, agriculture and forestry plant protection, electric power patrol inspection, land resource survey, and similar applications, it is of great significance to accurately remove haze, fog and smoke from remote sensing images (RSIs) for target detection, target tracking and UAV detection. For simplicity, the term dehazing is used uniformly to denote the removal of haze, fog and smoke.

In the image dehazing task, the following expression is widely used to describe the hazy image as^1–3^:

1 $$I\left(x\right)=J\left(x\right)t\left(x\right)+A\left(1-t\left(x\right)\right)$$

where $I(x)$, $J(x)$, *A* and* t* denote the hazy image, the haze-free image, the global atmospheric light, and the transmission map, respectively. Single image dehazing is a challenging problem, which is under-constrained due to the unknown depth information. At present, numerous dehazing algorithms from several directions have been proposed.

Early prior-based approaches have been demonstrated to be effective. Using Eq. (1), $A$ and $t$ must be accurately estimated to restore clear images. One of the most representative is the dark channel prior (DCP) method^4^ to determine the mapping relationship between clear images and atmospheric physical models, which is a relatively stable dehazing algorithm. However, the dehazing effect in large white areas tends to produce large deviations. Therefore, several researchers use data-driven deep learning approaches^5,6^ to estimate the intermediate parameters of atmospheric scattering model and construct a mapping relationship from the hazy image to the intermediate parameters. These deep learning algorithms are based on the atmospheric scattering model. Although they have greatly improved in the sky region and are visually more effective than traditional methods, the models are highly complex and vulnerable to the limitations of atmospheric lighting and scene changes, resulting in poor real-time performance and darkened brightness of the restored image. To address these problems, several algorithms directly predict the latent haze-free images in an end-to-end manner. Huang et al.^7^ proposed a conditional generative adversarial network that uses RGB and SAR images for dehazing. Mehta et al.^8^ developed SkyGAN specifically for removing haze in aerial images, addressing the challenge of limited hazy hyperspectral aerial image datasets.

In recent years, Vision Transformer (ViT)^9^ has excelled in high-level vision tasks, focusing on modeling long-term dependencies in data. However, earlier ViT and Pyramid Vision Transformer (PVT)^10^ were over-parameterized and computationally expensive. Thus, Liang et al.^11^ were inspired by Swin-Transformer^12^ and proposed SwinIR consisting of several Residual Swin Transformer Blocks (RSTB), each with several Swin Transformer layers and a residual connection. Uformer^13^ introduced a novel locally-enhanced window (LeWin) Transformer block and a learnable multi-scale restoration modulator in the form of a multi-scale spatial bias to adjust features in multiple layers of the Uformer decoder. Dong et al.^14^ proposed TransRA, a two-branch neural network fused with transformer and residual attention, to recover fine details of dehazing RSIs. Song et al.^15^ proposed Dehazeformer based on Swin-Transformer^12^ and U-Net^16^, modifying the standardization layer, activation function, and spatial information aggregation scheme, and introducing soft constraints using a weak prior. The Dehazeformer has shown superior performance compared to previous methods on SOTS indoor datasets, while being more efficient with fewer parameters and lower computational costs. However, it is difficult to obtain sufficient paired hazy RSI datasets due to natural conditions and equipment limitations. When the training samples are small and contain dense haze images, the Dehazeformer performs poorly in RSIs dehazing.

To sum up, in RSIs dehazing tasks, both local and global features are important, and traditional image dehazing methods rely on sound theoretical foundations that can guide network learning. Thus, we have designed a new RGB remote sensing image dehazing model (GTMNet) based on Dehazeformer by reconstructing the model architecture and combining DCP into the proposed network. Due to the down-sampling operations in the encoder of the Dehazeformer, the compressed spatial information may not be effectively retrieved by the decoder of the Dehazeformer. Therefore, we use the strengthen-operate-subtract (SOS) strategy in the decoder to retrieve more compressed information and gradually restore latent haze-free images in this work. We also compare several advanced dehazing models with GTMNet and verify the applicability of the proposed model. For this paper, the main contributions are as follows: (1) A novel hybrid architecture is proposed, which is based on CNN and ViT, and combines the DCP. Compared with other referenced models, it provides better PSNR and SSIM; (2) The transmission map optimized by guided filtering and a linear transformation is smoothly introduced into the model through the spatial feature transform (SFT) layer, enabling better estimation of the haze thickness in the image and thus improving performance; (3) To gradually refine the restored image in the feature recovery module, the SOS boosted module is combined into the image dehazing task via a skip connection.

## Proposed method

This section presents the details of GTMNet. First, we introduce the DCP. Then we estimate the transmission map. Finally, we describe the details of SFT layer, SOS boosted module and SK fusion module.

### Dark channel prior

He et al.^4^ conducted statistical analysis on non-sky regions of more than 5,000 haze-free outdoor images, and found that there are often some pixels with very low values in at least one color channel. Formally, the dark primary color of the haze-free image $J(x)$ is defined as:

2 $${J}^{dark}\left(x\right)={}_{c\in \left\{r,g,b\right\}}{}^{min }\left({}_{y\in \Omega \left(x\right)}{}^{min }\left({J}^{c}\left(y\right)\right)\right)$$

where *c* represents a channel among R, G, and B channels; *Ω*(*x*) is a local square centered at *x*; ${J}^{c}$ represents a certain color channel of $J$. The observation shows that, if $J$ is a haze-free outdoor image, except for the sky region, the pixel value of ${J}^{dark}$ tends to be 0. The above statistical observation is called the DCP or the dark primary color prior.

### Estimation of transmission map

To obtain a clear haze-free image $J$ in Eq. (1), it is necessary to solve *A* and* t*. Equation (1) can be rewrite as:

3 $$J(x)=A+\frac{I(x)-A}{t(x)}$$

According to the DCP, the dark channel of a haze image approximates the haze denseness well. Therefore, He et al.^4^ picked the top 0.1% brightest pixels in the dark channel of the hazy image. Among these pixels, the pixel with the highest intensity in the input image *I* is selected as the atmospheric light.

Assuming that the transmission in a local patch* Ω*(*x*) is constant, the patch’s transmission $\widehat{t}\left(x\right)$ can be defined as:

4 $$\widehat{t}\left(x\right)=1-{}_{y\in \Omega \left(x\right)}{}^{min }\left({}_{c }{}^{min}\left(\frac{{I}^{c}\left(y\right)}{{A}^{c}}\right)\right)$$

As mentioned in the literature^4^, even if the weather is clear, distant objects are more or less affected by haze, so the authors control the degree of haze by introducing a factor* ω* of [0,1] to give a sense of depth of field. The specific expression is:

5 $$\widehat{t}\left(x\right)=1-\omega {}_{y\in \Omega \left(x\right)}{}^{min }\left({}_{c }{}^{min}\left(\frac{{I}^{c}\left(y\right)}{{A}^{c}}\right)\right)$$

where *ω* is usually taken as 0.95.

Due to the local assumptions, the estimated transmission map $\widehat{t}\left(x\right)$ will exhibit block effects. In traditional image dehazing methods, $\widehat{t}\left(x\right)$ is usually refined using the soft matting method, guided filtering, or fast-guided filtering. Although the soft matting method can achieve good results, the edge information of the object is weak and it is time-consuming. Therefore, we use a fast-guided filter for optimization^17^, in which the filter window radius is set to 60 and the regularization parameter *e* is 0.0001.

**Figure 1:**
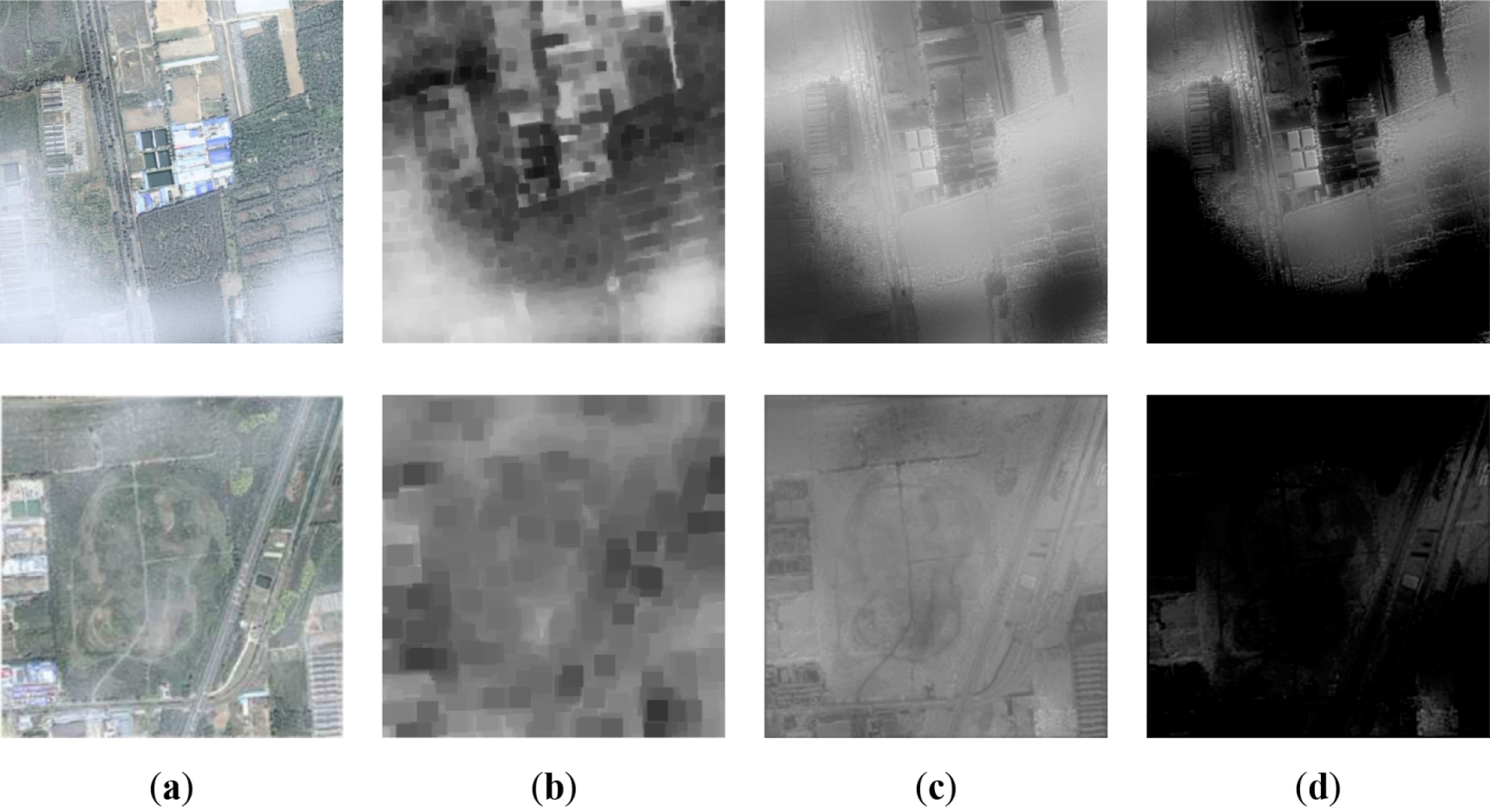
Results of transmission maps on SateHaze1k Dataset: (**a**) Input images; (**b**) Dark channel maps; (**c**) The transmission maps optimized by fast-guided filter; (**d**) The guided transmission maps.

Figure 1 shows the relevant results of transmission maps on the SateHaze1k dataset. We find that the transmission map optimized by the fast-guided filter in Fig. 1c can objectively estimate the hazy distribution of the input image. However, introducing the DCP in this paper aims to estimate the haze concentration. As shown in Fig. 1d, to highlight the haze thickness in the image, we used a linear transformation to enhance the optimized transmission map *t* and defined it as the guided transmission map (GTM) *t*1, which can be formulated as:

6 $$t1=2\times \left(t-0.5\right)$$

### GTMNet

**Figure 2:**
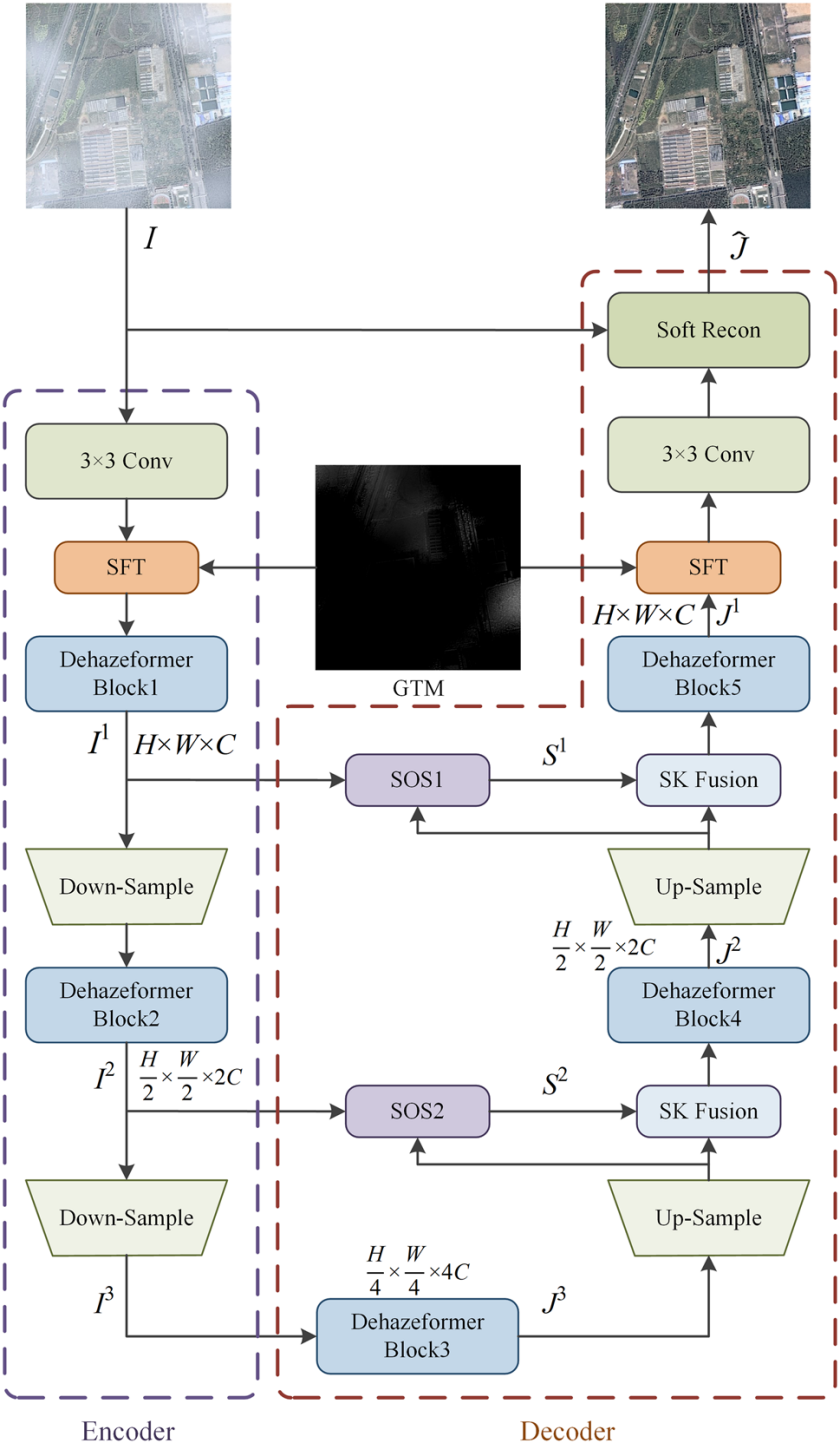
The overall architecture of proposed GTMNet.

As shown in Fig. 2 and Table 1, the proposed network GTMNet is based on Dehazeformer, but incorporates SFT layers^18^ and SOS boosted modules. SFT layers integrate the GTM into GTMNet, which can effectively fuse the features of the GTM and the input image to more accurately estimate the haze thickness in the input image. SOS boosted modules can restore clear images iteratively. At the end of the decoder, a soft reconstruction layer is used to estimate the haze-free image $\widehat{J}$.

**Table 1:** The architecture details of the proposed method (*Up* up-sampling factor, *Channel* number of input and output channels per block, *In and Out* spatial resolution of input and output per block, *Input* input per block).

Encoder						
Block	Filter size	Stride	Channel	In	Out	Input
3 × 3 conv	3 × 3	1	3/24	(H, W)	(H, W)	Hazy image
SFT	3 × 3	1	(24, 1)/24	(H, W)	(H, W)	F(3 × 3 conv), GTM
Dehazeformer Block1	–	–	24/24	(H, W)	(H, W)	F(SFT)
Down-Sample	3 × 3	2	24/48	(H, W)	(H/2, W/2)	F(Dehazeformer Block1)
Dehazeformer Block2	–	–	48/48	(H/2, W/2)	(H/2, W/2)	F(Down-Sample)
Down-Sample	3 × 3	2	48/96	(H/2, W/2)	(H/4, W/4)	F(Dehazeformer Block2)

Decoder						
Block	Filter size	Up	Channel	In	Out	Input
Dehazeformer Block3	–	–	96/96	(H/4, W/4)	(H/4, W/4)	F(Down-Sample)
Up-Sample	3 × 3	2	96/48	(H/4, W/4)	(H/2, W/2)	F(Dehazeformer Block3)
SOS2	3 × 3	1	(48,48)/48	(H/2, W/2)	(H/2, W/2)	F(Up-Sample), F(Dehazeformer Block2)
SK Fusion	–	–	(48,48)/48	(H/2, W/2)	(H/2, W/2)	F(SOS2), F(Up-Sample)
Dehazeformer Block4	–	–	48/48	(H/2, W/2)	(H/2, W/2)	F(SK Fusion)
Up-Sample	3 × 3	2	48/24	(H/2, W/2)	(H, W)	F(Dehazeformer Block4)
SOS1	3 × 3	1	(24,24)/24	(H, W)	(H, W)	F(Up-Sample), F(Dehazeformer Block1)
SK Fusion	–	–	(24,24)/24	(H, W)	(H, W)	F(SOS1), F(Up-Sample)
Dehazeformer Block5	–	–	24/24	(H, W)	(H, W)	F(SK Fusion)
SFT	3 × 3	1	(24,1)/24	(H, W)	(H, W)	F(Dehazeformer Block5),GTM
3 × 3 conv	3 × 3	1	24/4	(H, W)	(H, W)	F(SFT)
Soft Recon	–	–	(4,3)/3	(H, W)	(H, W)	F(3 × 3 conv), hazy image

#### SFT layer

**Figure 3:**
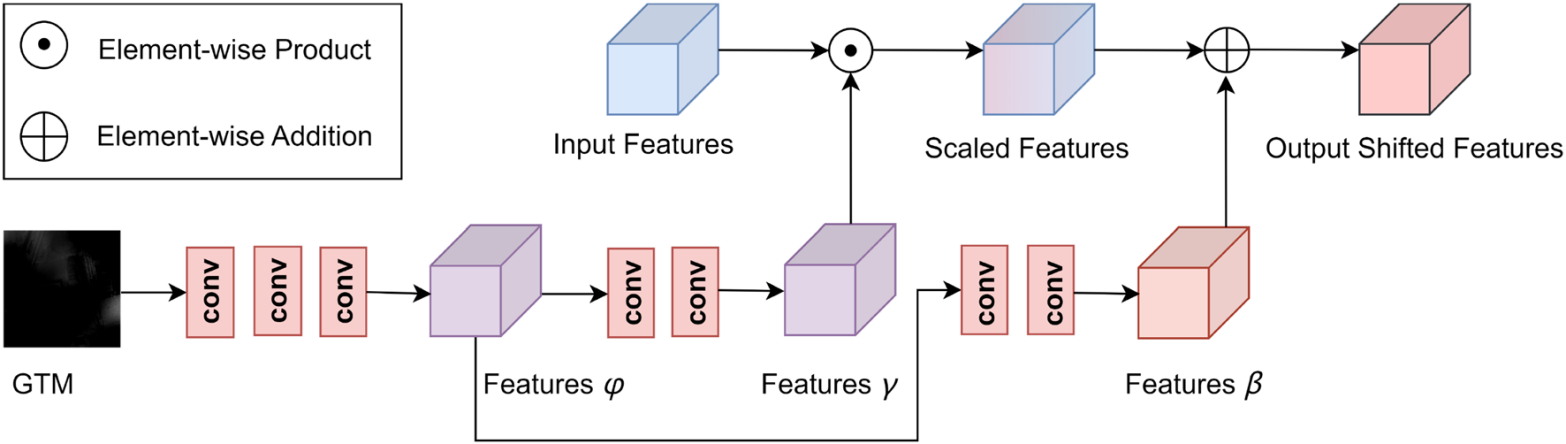
The structure of SFT Layer.

The SFT layer is first applied in super-resolution tasks^18^. It is parameter-efficient and can be easily introduced to existing dehazing network structures with strong extensibility. As shown in Fig. 3, we use the GTM *t*1 as the additional input of the SFT layer, which first applies three convolutional layers to extract the conditional maps *φ* from the GTM; then the conditional maps *φ* is input to the other two convolutional layers to predict the modulation parameters *γ* and *β*, respectively; finally, the transformation is carried out by scaling and shifting feature maps of a specific layer, and we can obtain the output shifted features by:

7 $$SFT(F|\gamma , \beta ) = \gamma \odot F\oplus \beta$$

where *F* is the feature maps with the same dimensions as *γ* and *β*, ⊙ is referred to the element-wise multiplication, i.e., Hadamard product, and ⊕ is the element-wise addition. Since the spatial dimensions are preserved, the SFT layer performs feature-wise manipulation and spatial-wise transformation. Since the size of each object is generally tiny in RSIs, obtaining local features becomes crucial. In this paper, we utilized SFT layers with shared parameters to compensate for the Transformer's limited ability to acquire local features.

#### SOS boosted module

**Figure 4:**
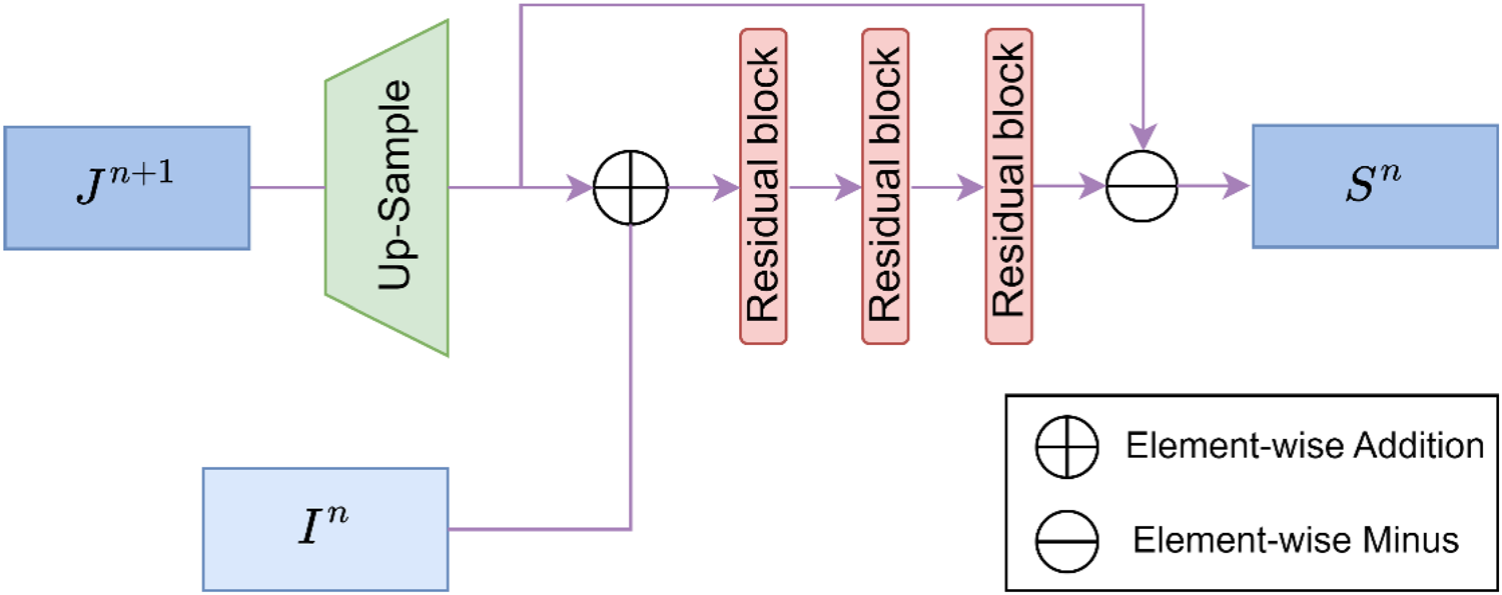
The structure of SOS boosted module.

The SOS boosting method^19^ has been mathematically proven to be effective for image denoising, which iteratively restores clear images. Dong et al.^20^ have verified a variety of optional SOS boosted modules, and the results show that the following boosted scheme has the best effect, as shown in Eq. (8):

8 $${S}^{n}={\mathcal{G}}_{{\theta }_{n}}^{n}({I}^{n}+Up{(S}^{n+1}))-{Up(S}^{n+1})$$

where $Up(.)$ denotes the upsampling operator using a pixel shuffle method^21^, ${S}^{n+1}$ represents the previous level feature, ${I}^{n}$ denotes the latent feature from the encoder, $({I}^{n}+Up{(S}^{n+1}))$ represents the strengthened feature, and ${\mathcal{G}}_{{\theta }_{n}}^{n}$ denotes the trainable refinement unit at the (*n*)*-th* level parameterized by ${\theta }_{n}$. According to the proposed architecture, Eq. (8) is written as Eq. (9):

9 $${S}^{n}={\mathcal{G}}_{{\theta }_{n}}^{n}({I}^{n}+Up{(J}^{n+1}))-Up{(J}^{n+1})$$

where ${J}^{n+1}$ denotes the feature from the Dehazeformer block of the decoder. The SOS boosted module consists of three residual blocks, as shown in Fig. 4.

#### SK fusion module

Song et al.^22^ designed a selective kernel (SK) Fusion module, which is inspired by SKNet^23^, to fuse multiple branches using channel attention. We use the SK Fusion module^22^ to fuse the SOS and decoder branches. Specifically, let two feature maps $x1$ and $x2$, a linear layer $f\left(.\right)$ is first used to project $x1$ to $\widehat{x}1$. Then a global average pooling $GAP\left(.\right)$, a Multilayer Perceptron $MLP\left(.\right)$, a softmax function and a split operation are used to obtain fusion weights, as shown in Eq. (10):

10 $$\left\{a1,a2\right\}=Split\left(Softmax\left(MLP\left(GAP\left(\widehat{x}1+x2\right)\right)\right)\right)$$

Finally, weights $\left\{a1,a2\right\}$ are used to fuse $\widehat{x}1$, $x2$ with an additional short residual via $y=a1\widehat{x}1+a2x2+x2$.

## Experiments

In this part, we first present datasets and the implementation details of GTMNet. Then, we evaluate our method on RS-Haze and SateHaze1k datasets. Finally, ablation studies and other comparative experiments are conducted to analyze the proposed approach.

### Datasets

*RS-Haze*^22^ is a synthetic hazy RSI dataset synthesized from 76 RSIs containing diverse topography with good weather conditions and 108 cloudy RSIs. All the images are downloaded from the Landsat-8 Level 1 data product on EarthExplorer. The final training set contains 51,300 RSI pairs, and the test set contains 2,700 RSI pairs with an image resolution of 512 × 512. Since the proposed method is optimized on the Dehazeformer model, the experimental setup is consistent with the Dehazeformer^22^. We train the model using L1 loss for 150 epochs, each of which is validated once. The images in the test set are the same as those in the verification set.

*SateHaze1k*^7^ is also a synthetic haze satellite remote sensing dataset, which uses Photoshop software as an auxiliary tool to generate rich, real and diverse hazy images. This dataset contains 1,200 RSI pairs, and each pair of images includes a hazy image and a real haze-free image. These images are divided into three haze image subsets: Thin Fog, Moderate Fog and Thick Fog, with an image resolution of 512 × 512. We select 320 pairs of images from each type of hazy image subset as the training set and 45 pairs of images as the test set. Each type of hazy image subset is trained and tested separately. Since the SateHaze1k dataset is small, we train GTMNet for 1000 epochs and verify it every ten epochs. Other experimental configurations are the same as those of the RS-Haze dataset.

### Implementation details

We provide four variants of GTMNet (-T, -S, -B and -L for tiny, small, basic, and large, respectively), implement the proposed network structure using the PyTorch framework, and train the model on an NVIDIA GeForce RTX3090. During training, images are randomly cropped to 256 × 256 patches. We set different mini-batch sizes for different variants, i.e., {32, 16, 8, 4} for {-T, -S, -B, -L}. The initial learning rate is set to {4, 2, 2, 1} × 10^–4^ for the variant {-T, -S, -B, -L}. We use the AdamW optimizer^24^ with a cosine annealing strategy^25^ to train the model, where the learning rate gradually decreases from the initial learning rate to {4, 2, 2, 1} × 10^–6^.

The proposed mechanism for GTMNet training is illustrated in Algorithm 1. All the learnable parameters in GTMNet are initialized using the truncated normal distribution strategy^26^.
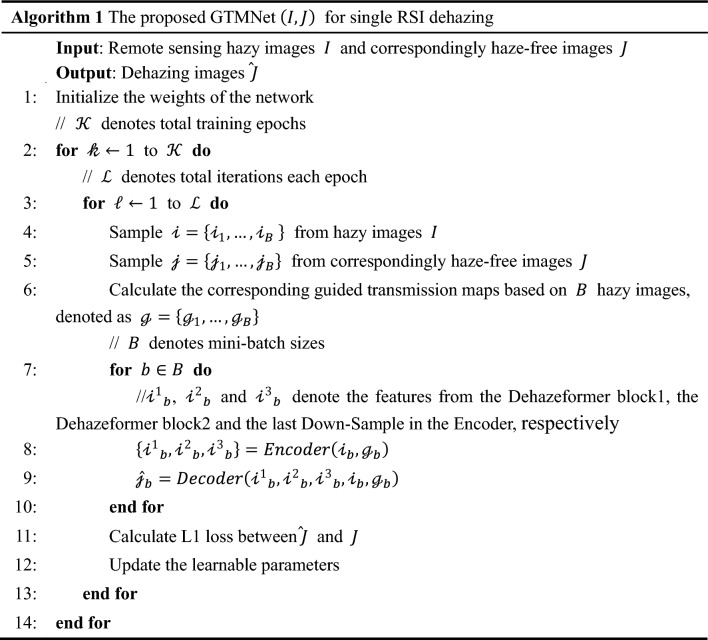


### Evaluation

#### Quantitative evaluation

We use Peak Signal to Noise Ratio (PSNR) and Structure Similarity Index Measurement (SSIM) as objective evaluation indicators, and compare the number of parameters between GTMNet and other methods, as shown in Tables 2 and 3, where bold indicates the optimal value and underline indicates the suboptimal value.

**Table 2:** Quantitative comparison of GTMNet-T with other methods on RS-Haze dataset.

Setting	PSNR/dB	SSIM	Params
DCP^4^	17.86	0.734	–
DehazeNet^5^	23.16	0.816	0.009 M
GCANet^27^	34.41	0.949	0.702 M
Dehazeformer-T^22^	**39.11**	**0.968**	0.686 M
GTMNet-T	38.99	**0.968**	0.798 M

**Table 3:** Quantitative comparison of GTMNet with other methods on SateHaze1k dataset.

Setting	Thin fog		Moderate fog		Thick fog		Params
	PSNR/dB	SSIM	PSNR/dB	SSIM	PSNR/dB	SSIM	
DCP^4^	13.15	0.725	9.78	0.574	10.25	0.585	–
DehazeNet^5^	19.75	0.895	18.12	0.855	14.33	0.706	0.009 M
Huang (SAR)^7^	24.16	0.906	25.31	0.926	**25.07**	0.864	–
SkyGAN^8^	25.38	0.925	25.58	0.904	23.43	0.893	–
TransRA^14^	25.20	0.930	26.50	0.947	22.73	0.875	–
Dehazeformer-T^22^	24.51	0.968	26.38	0.969	22.02	0.932	0.686 M
Dehazeformer-S^22^	24.60	0.968	26.52	0.971	22.11	0.933	1.283 M
Dehazeformer-B^22^	25.18	0.971	26.74	**0.973**	22.23	0.934	2.514 M
Dehazeformer-L^22^	**25.89**	**0.974**	**27.23**	**0.973**	23.14	**0.942**	25.44 M
GTMNet-T	25.14	0.970	26.73	0.971	22.31	0.935	0.798 M
GTMNet-S	24.72	0.967	26.61	0.972	22.29	0.935	1.396 M
GTMNet-B	24.89	0.970	27.22	**0.973**	23.02	0.939	2.629 M
GTMNet-L	25.52	0.972	27.18	**0.973**	22.96	0.940	25.87 M

#### RS-Haze dataset

Due to the equipment limitations, only testing and training are conducted on -T. We compare the proposed method with four other classical dehazing algorithms. As shown in Table 2, the PSNR of our method is slightly lower than that of Dehazeformer-T, while the SSIM of both is the same. Since the proposed architecture has more parameters, it is easier to overfit, resulting in poor generalization performance.

#### SateHaze1k dataset

We compare the proposed method with DCP^4^, DehazeNet^5^, Huang (SAR)^7^, SkyGAN^8^, TransRA^14^ and Dehazeformer^22^, and the results are shown in Table 3. The PSNR and SSIM of GTMNet-T on the three sub-datasets are better than that of Dehazeformer-T^22^, especially, the PSNR on Thin Fog is improved by nearly 2.6%, and the SSIM is increased from 0.968 to 0.970. On Moderate Fog, the PSNR and SSIM of GTMNet-B reach 27.22 dB and 0.973, respectively, an increase of 7.2% and 7.6% compared to SkyGAN^8^. On Thick Fog, although the PSNR of GTMNet-B is lower than that of Huang (SAR)^7^ and SkyGAN^8^, the SSIM metric improves by 8.7% and 5.2%, respectively, compared to the two algorithms. On the three sub-datasets, GTMNet-T achieves better PSNR and SSIM scores than TransRA^14^, with a significant improvement in PSNR performance.

As shown in Table 3, combined with the quantitative comparison results above, the proposed model is still lightweight, although the parameters have increased slightly. On Moderate Fog and Thick Fog sub-datasets, GTMNet-B performs comparably to Dehazeformer-L, but with only 0.1 times the number of parameters. However, the performance of GTMNet-L is inferior to that of Dehazeformer-L, which may be caused by two aspects: Firstly, the increased parameter quantity of GTMNet-L makes it more prone to overfitting; Secondly, the generalization ability of GTMNet-L is reduced due to the small dataset.

#### Qualitative evaluation

**Figure 5:**
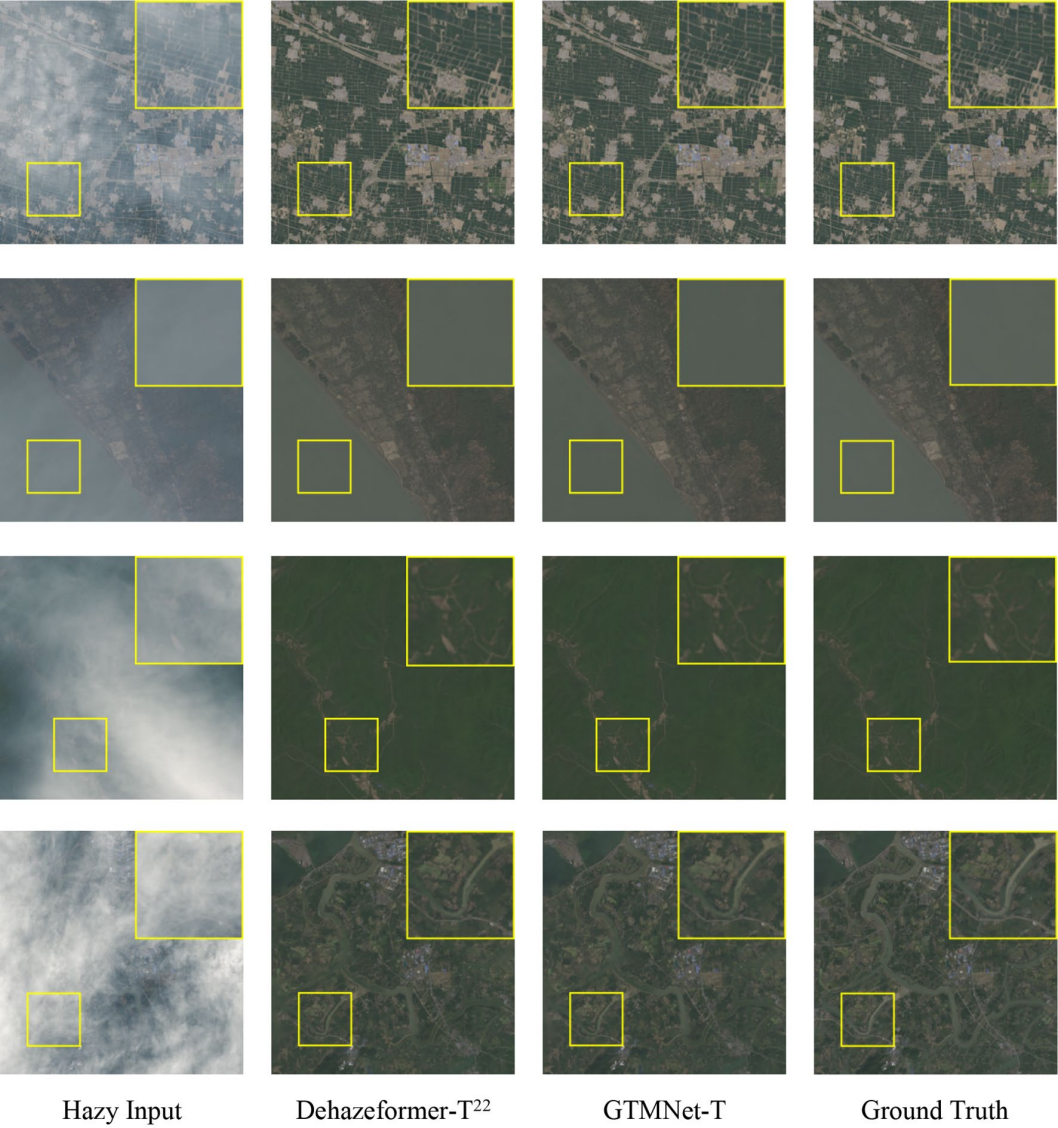
Qualitative comparison of image dehazing methods on RS-Haze dataset.

A qualitative comparison of related methods was performed on the RS-Haze and SateHaze1k datasets. Since Song et al.^22^ has compared the existing advanced dehazing image methods on RS-Haze dataset, we only present the dehazed images of GTMNet-T and Dehazeformer-T here. As shown in Fig. 5, there is little visual difference between GTMNet-T and Dehazeformer-T on the RS-Haze images, both showing clarity, rich feature information, realistic colours and a sense of hierarchy.

**Figure 6:**
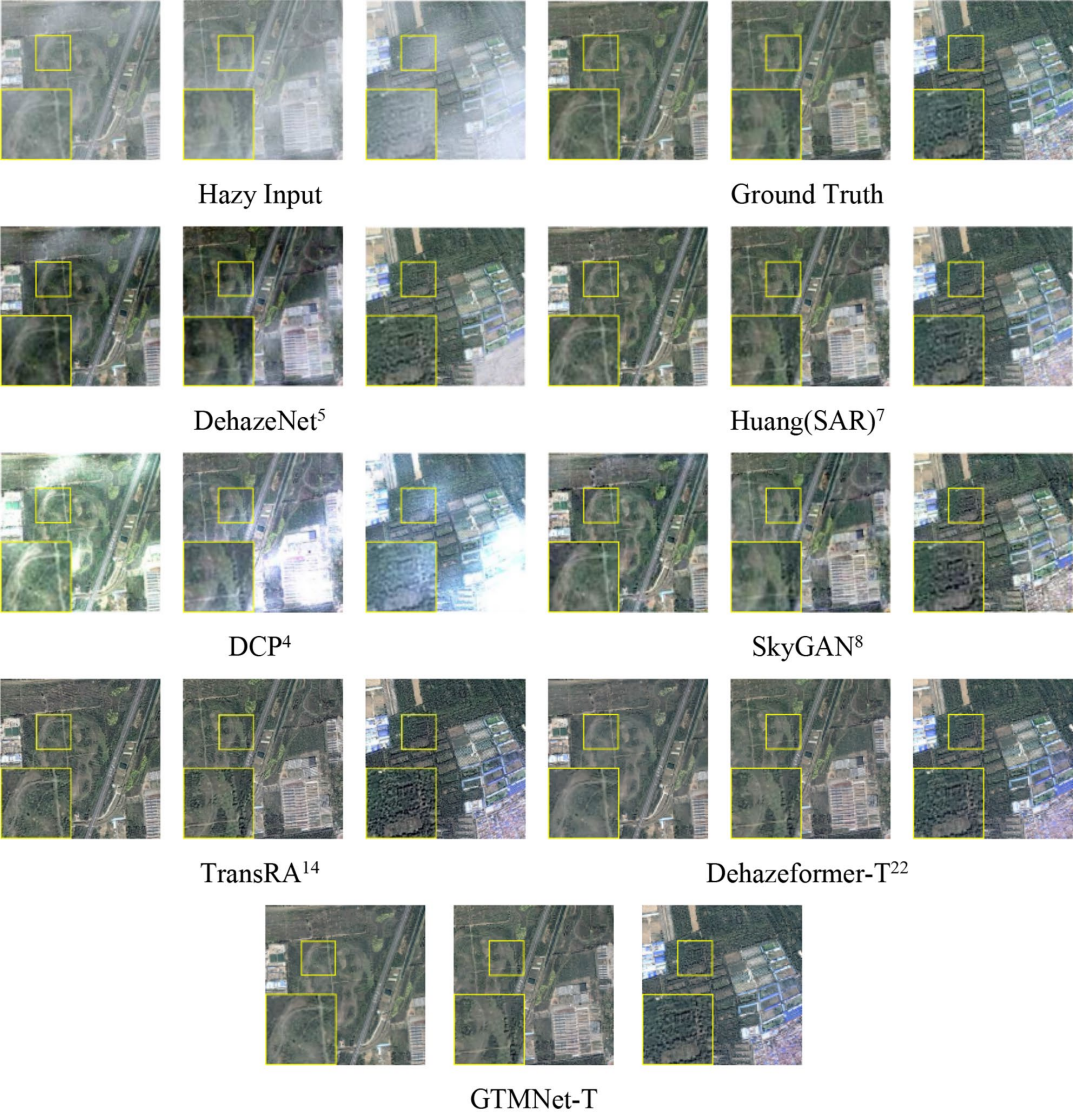
Qualitative comparison of image dehazing methods on SateHaze1k dataset.

On SateHaze1k dataset, we present the qualitative comparison results of the GTMNet and state-of-the-art methods. The hazy input images include farmland, roads, buildings and vegetation, as shown in Fig. 6. We found that the DCP^4^ method failed, possibly due to the similarity between the colors of the atmospheric light and the object. Although the method of Huang (SAR)^7^ can remove haze, the ground feature information of the restored image in the dense haze area is not rich enough, and the building details are severely weakened. In general, both DehazeNet^5^ and SkyGAN^8^ failed to completely remove the haze (as shown in the processing result of the first hazy image in Fig. 6), resulting in unnatural color of the image and weak recovery ability for detailed information. Dehazeformer-T^22^ and GTMNet-T solve the problem of incomplete image dehazing. However, for areas with thick haze or cloud haze, the Dehazeformer algorithm suffers from serious color distortion. GTMNet improves not only the problem of image color deviation but also the sharpness.

### Ablation study

In this part, we perform ablation studies on the proposed model structure to analyze the factors that may influence the results. In these studies, except for different subjects, the other strategies are the same in each group of experiments.

#### The effects of different components on the model performance

To study the influence of different components on the image dehazing effect, we take Dehazeformer-T^22^ as the baseline model and conduct ablation experiments on different components on SateHaze1k dataset^7^.

As shown in Table 4, D-SOS-T refers to adding the SOS module to Dehazeformer-T. According to Table 5, we found that the PSNR and SSIM indicators of the three sub-datasets have been significantly improved, verifying the effectiveness of the SOS module in the image dehazing task. D-GTM-T indicates the introduction of the GTM as a prior into Dehazeformer-T through two SFT layers. The location of the SFT layer is shown in Fig. 9b. According to Table 5, the performance of adding only a prior GTM to Dehazeformer-T without using the SOS boosted strategy is better than that of Dehazeformer-T on Moderate Fog, but the effect is poor on Thin Fog and Thick Fog. We believe this is because the method for obtaining GTM is based on statistics for ordinary images, which have a large gap between RSIs and ordinary images. Traditional prior methods are more effective in uniform haze images.

**Table 4:** Ablation models of different components.

Models	SOS	Number of SFT layers	GTM
Dehazeformer-T		0	
D-SOS-T	✓	0	
D-GTM-T		2	✓
GTMNet-T	✓	2	✓

**Table 5:** Quantitative comparison of different components ablation models on SateHaze1k dataset.

Setting	Thin fog		Moderate fog		Thick fog		Params
	PSNR/dB	SSIM	PSNR/dB	SSIM	PSNR/dB	SSIM	
Dehazeformer-T	24.51	0.968	26.38	0.969	22.02	0.932	0.686 M
D-SOS-T	24.84	0.969	**27.09**	0.971	22.24	0.933	0.766 M
D-GTM-T	24.15	0.967	26.81	**0.973**	21.99	0.929	0.720 M
GTMNet-T	**25.14**	**0.970**	26.73	0.971	**22.31**	**0.935**	0.798 M

Bold indicates the optimal value and underline indicates the suboptimal value.

**Figure 7:**
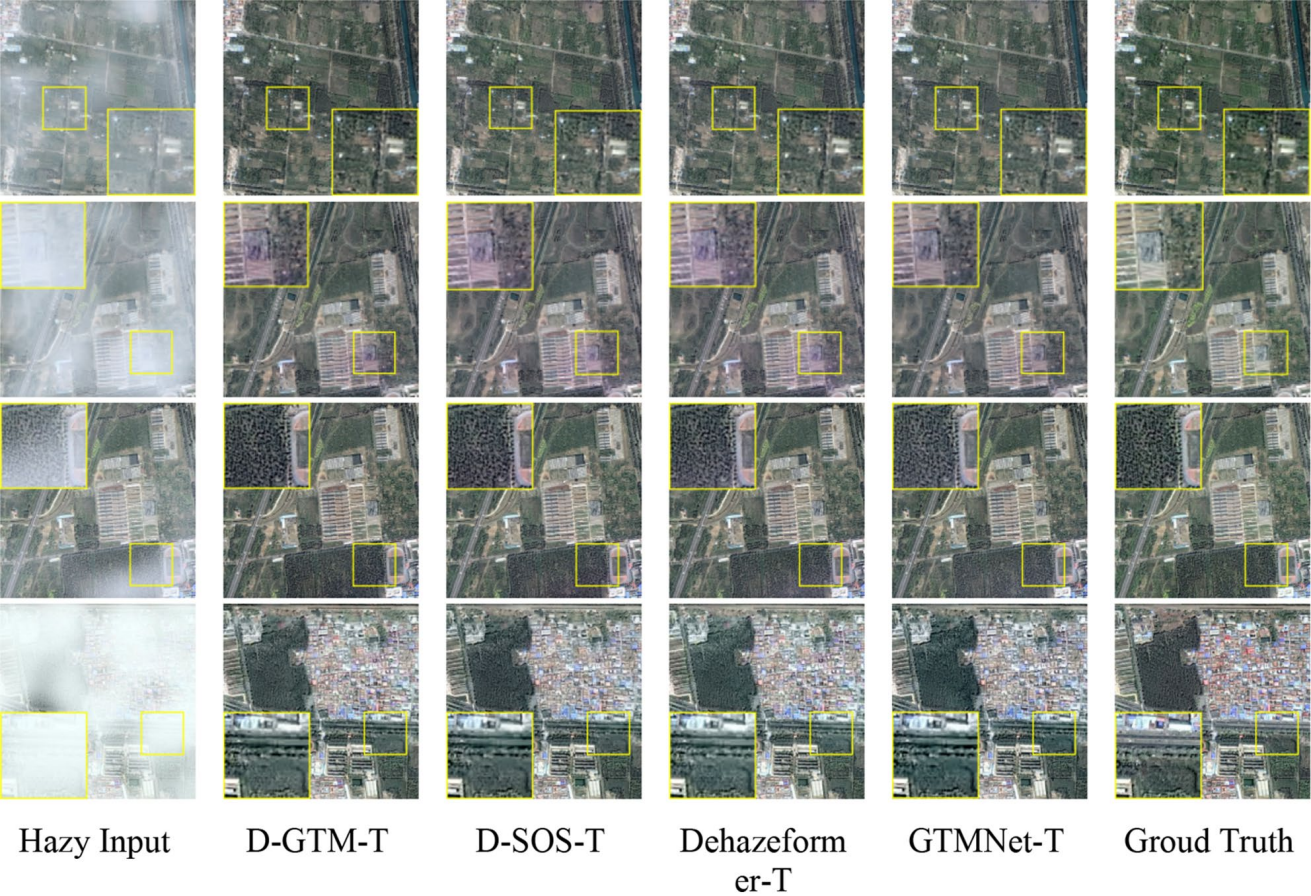
Qualitative comparison of different components ablation models on SateHaze1k dataset.

As shown in Fig. 7, the haze-free images generated by Dehazeformer-T, D-SOS-T, and D-GTM-T all show building distortion. Among all the methods, the dehazing effect of GTMNet is the best, which can ensure the clarity of the restored image and better restore the color of the image. On Thin Fog and Thick Fog sub-datasets, the PSNR and SSIM indicators increase more when the two components are used together than when used separately.

#### The effects of different inputs of SOS1 module on the model performance

According to Eq. (8–9), we designed two different ablation models D-SOS-T and D-SOS1-T on SateHaze1k dataset. The specific configuration is shown in Table 6. According to Table 7, if ${S}^{2}$ is directly upsampled and input to SOS1 (Fig. 2), compared with D-SOS-T, PSNR decreases from 27.09 to 26.77 dB, and the value of SSIM remains unchanged on Moderate Fog. In addition, compared with Dehazeformer-T, PSNR and SSIM increase from 26.38 dB and 0.969 to 26.77 dB and 0.971, respectively.

**Table 6:** Ablation models of different inputs to the SOS1 module.

Models	SOS	SOS1 Inputs
Dehazeformer-T		
D-SOS-T	✓	$Up({J}^{2})$
D-SOS1-T	✓	${Up(S}^{2}$)

**Table 7:** Quantitative comparison of ablation models with different inputs to the SOS1 module on SateHaze1k dataset.

Setting	Thin fog		Moderate fog		Thick fog		Params
	PSNR/dB	SSIM	PSNR/dB	SSIM	PSNR/dB	SSIM	
Dehazeformer-T	24.51	0.968	26.38	0.969	22.02	0.932	0.686 M
D-SOS-T	**24.84**	**0.969**	**27.09**	**0.971**	**22.24**	0.933	0.766 M
D-SOS1-T	24.80	0.967	26.77	**0.971**	**22.24**	**0.934**	0.767 M

Bold indicates the optimal value and underline indicates the suboptimal value.

**Figure 8:**
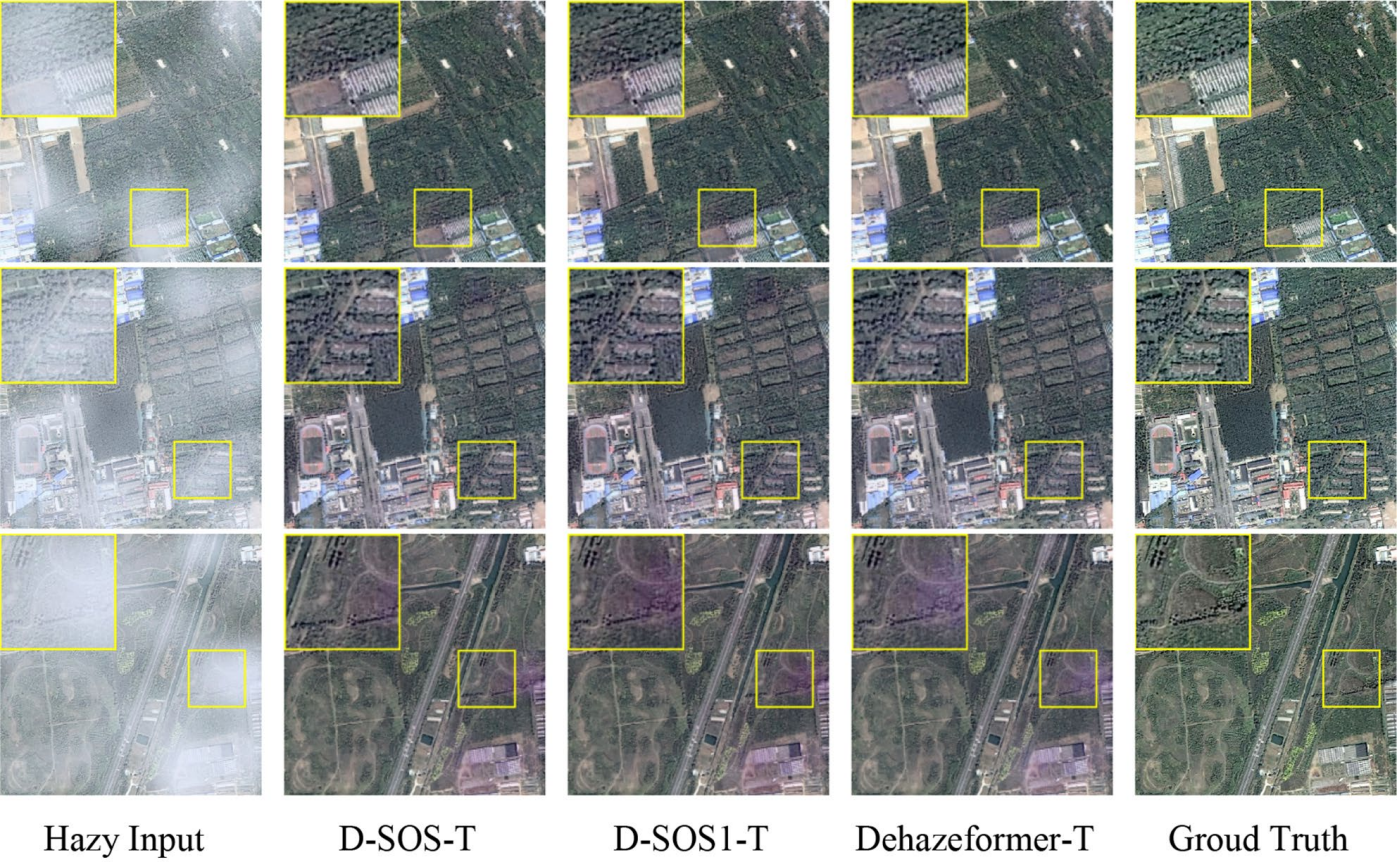
Qualitative comparison of ablation models with different inputs to the SOS1 module on SateHaze1k dataset.

As seen in Fig. 8, there is very little visual difference between the dehazed images of D-SOS-T and D-SOS1-T. In the dense haze area, the color distortion is severe and the edge detail is lost, as shown in the results of the third hazy image in Fig. 8. To sum up, $Up({J}^{2})$ is set as the input of SOS1 module.

#### The effects of SFT layer and GTM on the model performance

**Figure 9:**
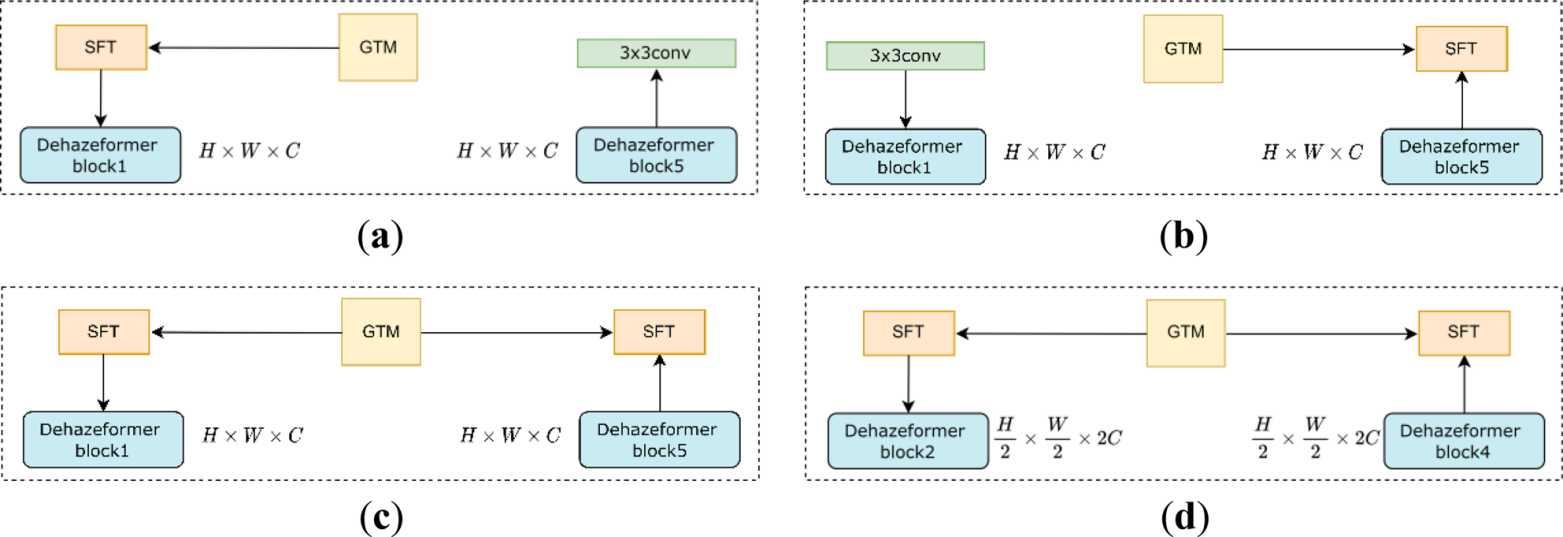
Position of SFT layers: (**a**) In front of Dehazeformer block1; (**b**) Behind Dehazeformer block5; (**c**) In front of Dehazeformer block1 and behind Dehazeformer block5; (**d**) In front of Dehazeformer block2 and behind Dehazeformer block4.

**Figure 10:**
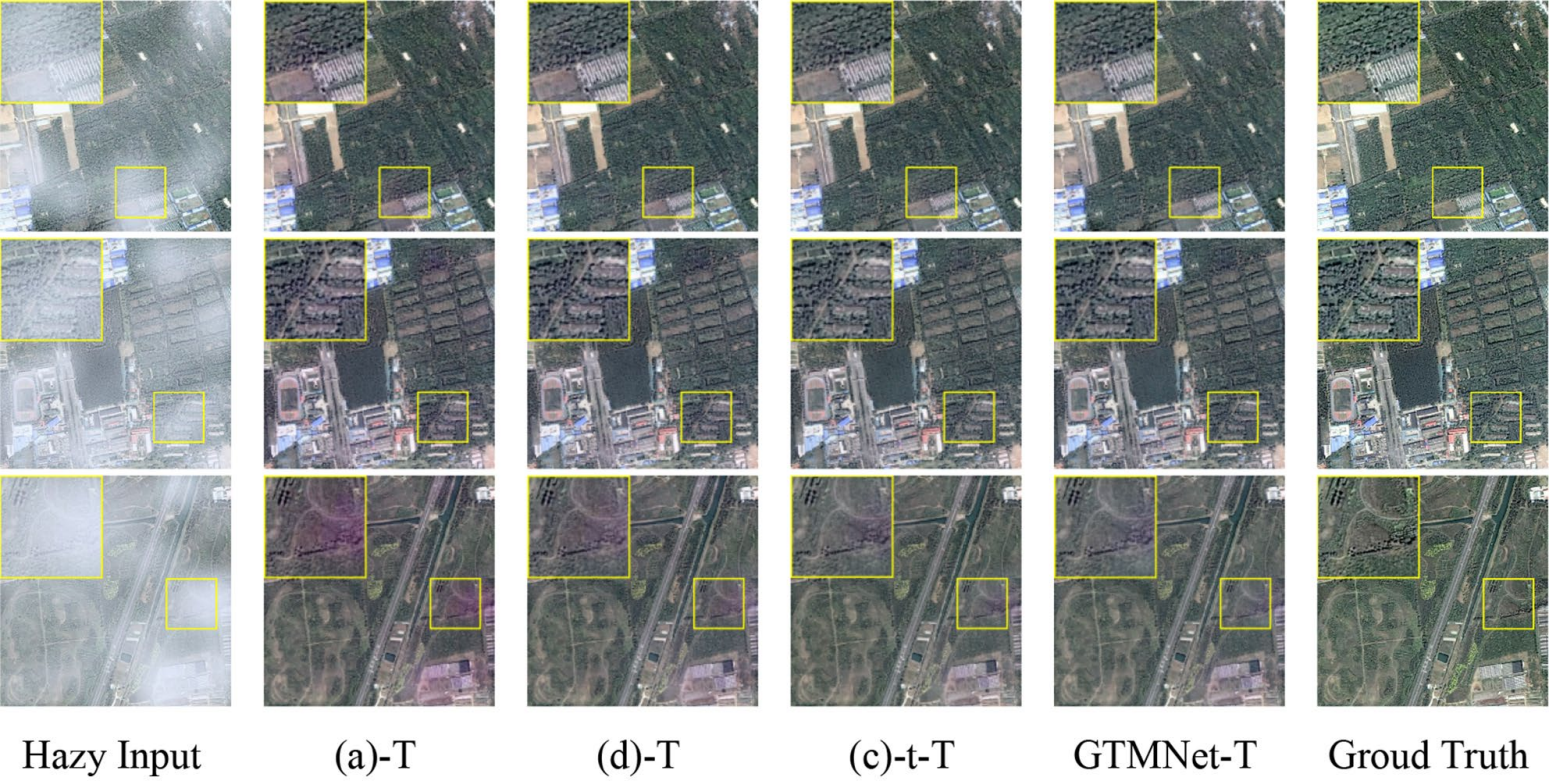
Qualitative comparison of ablation models of SFT layer and GTM on SateHaze1k dataset.

According to the structure of the model, the position of SFT layers can be categorized into four situations (as shown in Fig. 9): (a) using only one SFT layer in front of Dehazeformer block1, (b) using only one SFT layer behind Dehazeformer block5, (c) using an SFT layer in front of Dehazeformer block1 and behind Dehazeformer block5, respectively (i.e., GTMNet), and (d) using an SFT layer in front of Dehazeformer block2 and behind Dehazeformer block4, respectively. As shown in Table 8, (d)-T has the highest PSNR and SSIM on Moderate Fog, but Table 9 indicates that GTMNet-B has a greater increase in PSNR and SSIM than (d)-B. Moreover, as seen from the comparison results in Fig. 10, the best dehazed result is achieved using GTMNet-T, with significantly improved image clarity and less severe image color distortion, especially in the third hazy image in Fig. 10.

**Table 8:** Quantitative comparison of ablation models of SFT layer and GTM on SateHaze1k dataset.

Setting	Thin fog		Moderate fog		Thick fog		Params
	PSNR/dB	SSIM	PSNR/dB	SSIM	PSNR/dB	SSIM	
Dehazeformer-T	24.51	0.968	26.38	0.969	22.02	0.932	0.686 M
(a)-T	24.82	0.968	26.92	0.972	22.23	0.934	0.798 M
(b)-T	24.92	0.969	26.96	0.972	22.41	**0.935**	0.798 M
(d)-T	**25.21**	0.969	**27.02**	0.972	**22.50**	**0.935**	0.868 M
(c)-t-T	24.91	0.969	26.79	0.972	21.78	0.929	0.798 M
GTMNet-T	25.14	**0.970**	26.73	0.971	22.31	**0.935**	0.798 M

Bold indicates the optimal value and underline indicates the suboptimal value.

**Table 9:** Quantitative comparison of ablation models and GTMNet with different variants on SateHaze1k dataset.

Setting	Thin fog		Moderate fog		Thick fog		Params
	PSNR/dB	SSIM	PSNR/dB	SSIM	PSNR/dB	SSIM	
(d)-S	24.84	0.969	26.55	0.972	22.08	0.933	1.465 M
(d)-B	24.65	0.968	26.39	0.972	22.00	0.929	2.698 M
GTMNet-S	24.72	0.967	26.61	0.972	22.29	0.935	1.396 M
GTMNet-B	**24.89**	**0.970**	**27.22**	**0.973**	**23.02**	**0.939**	2.629 M

Bold indicates the optimal value and underline indicates the suboptimal value.

Based on the results shown in Table 8, we conclude that adding GTM to both the encoder and decoder has a superior effect on removing haze from the Thin Fog RSIs, and adding GTM solely to the decoder has a better effect on removing haze from the Moderate Fog and Thick Fog RSIs. We believe that the effectiveness of GTM is not only related to the thickness of haze, but also depends on the presence or absence of SOS boosted modules.

Different transmission maps can impact the dehazing performance of a model. In our experiment, we utilized two types of transmission maps: the transmission map optimized solely by guided filtering, named (c)-t-T, and the GTM obtained by optimizing the estimated transmission map via guided filtering and subsequently applying a linear transformation to it, which was used in GTMNet. As shown in Table 8, the GTM leads to higher PSNR and SSIM indicators on both Thin Fog and Thick Fog compared to the transmission map optimized solely by guided filtering. Moreover, the subjective visual evaluation and objective quantitative metrics results demonstrate that GTM is also suitable for local dense haze images and yields a remarkable dehazing effect.

#### The effects of initial learning rate on the model performance

According to the training method in Dehazeformer^22^, the initial learning rate of the model decreases as the batch size decreases. Following the linear scaling rule, the initial learning rate of GTMNet-B should be 1 × 10^–4^. We performed ablation experiments on three sub-datasets and found that if we reduced the initial learning rate on GTMNet-B, as shown in Table 10, the values of PSNR and SSIM generally decreased significantly, so we kept the initial learning rate constant, i.e., 2 × 10^–4^, even if we reduced the batch size of an iteration on -B.

**Table 10:** Quantitative comparison of different initial learning rates in GTMNet-B model.

Setting	Thin Fog		Moderate Fog		Thick Fog		Params
	PSNR/dB	SSIM	PSNR/dB	SSIM	PSNR/dB	SSIM	
GTMNet-B	24.89	0.970	27.22	0.973	23.02	0.939	2.629 M
Lr → 1 × 10^–4^	25.38	0.971	26.84	0.973	22.02	0.934	2.629 M

### Quantitative comparison of real-world images

**Figure 11:**
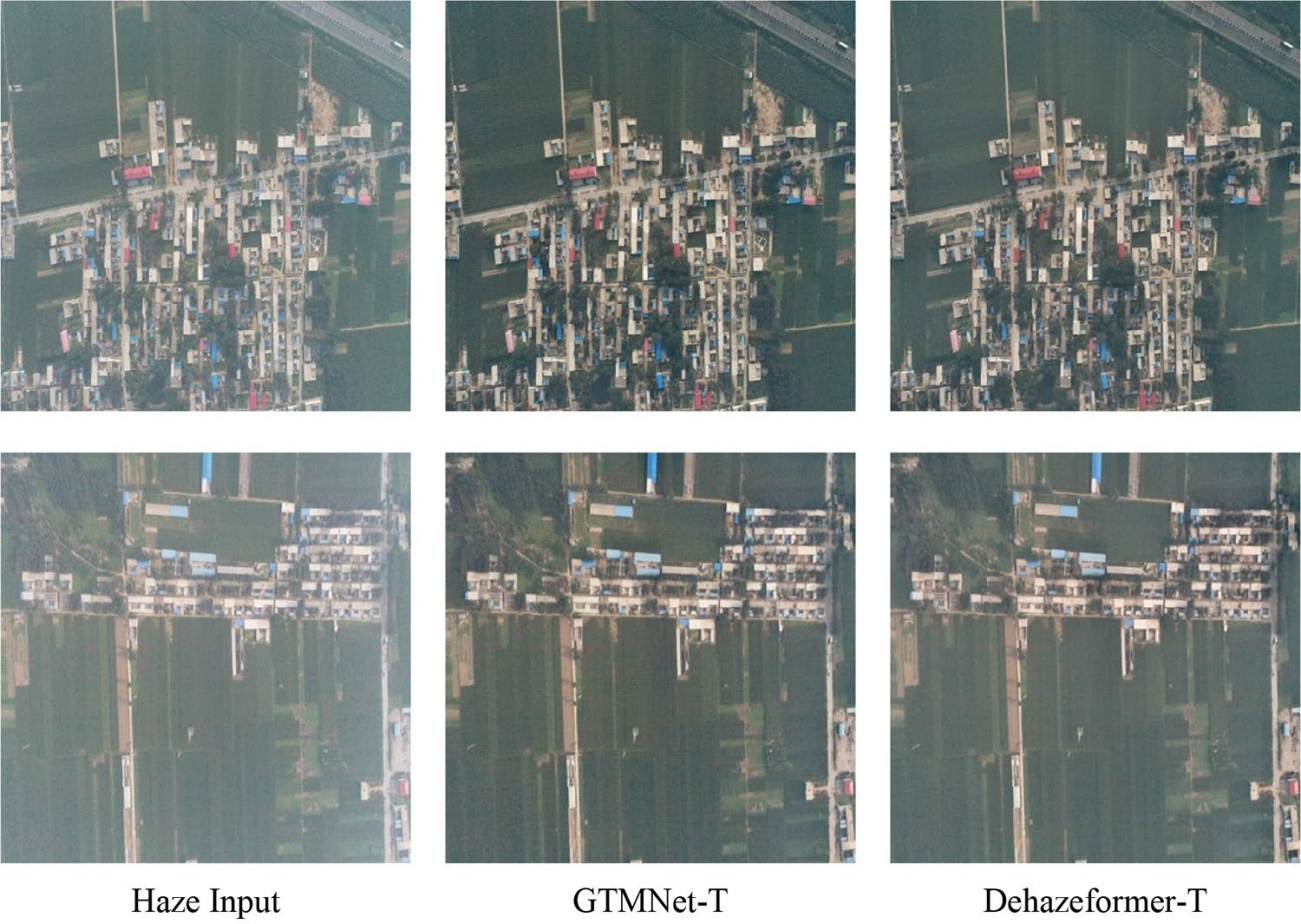
Quantitative comparison of Dehazeformer and GTMNet for real-world images. The hazy inputs are acquired by a DJI-Phantom 4 Pro.

In order to evaluate the generalization ability of the GTMNet, we select two real-world unmanned aerial hazy RSIs for testing. Overall, the Dehazeformer method is suboptimal; therefore, we only compare the results of GTMNet-T and Dehazeformer-T in this part and use the -T model trained on Moderate Fog to test the two real-world haze images. Figure 11 shows little visual difference between the processing results obtained by the proposed algorithm and Dehazeformer-T. Both methods produce clear, rich ground information, and realistic colors, suggesting that both algorithms are suitable for hazy remote sensing images in the real world. We have included additional visual comparisons in Supplementary Material to showcase the performance of our method on real-world images (Supplementary material).

### The impact of dehazing results on subsequent tasks

Hazy images suffer from problems like low contrast, low saturation, detail loss, and color deviation, which seriously affect image analysis tasks, such as classification, positioning, detection, and segmentation. Therefore, in such cases, dehazing is crucial for generating images with good perceptual quality and improving the performance of subsequent computer vision tasks.

**Figure 12:**
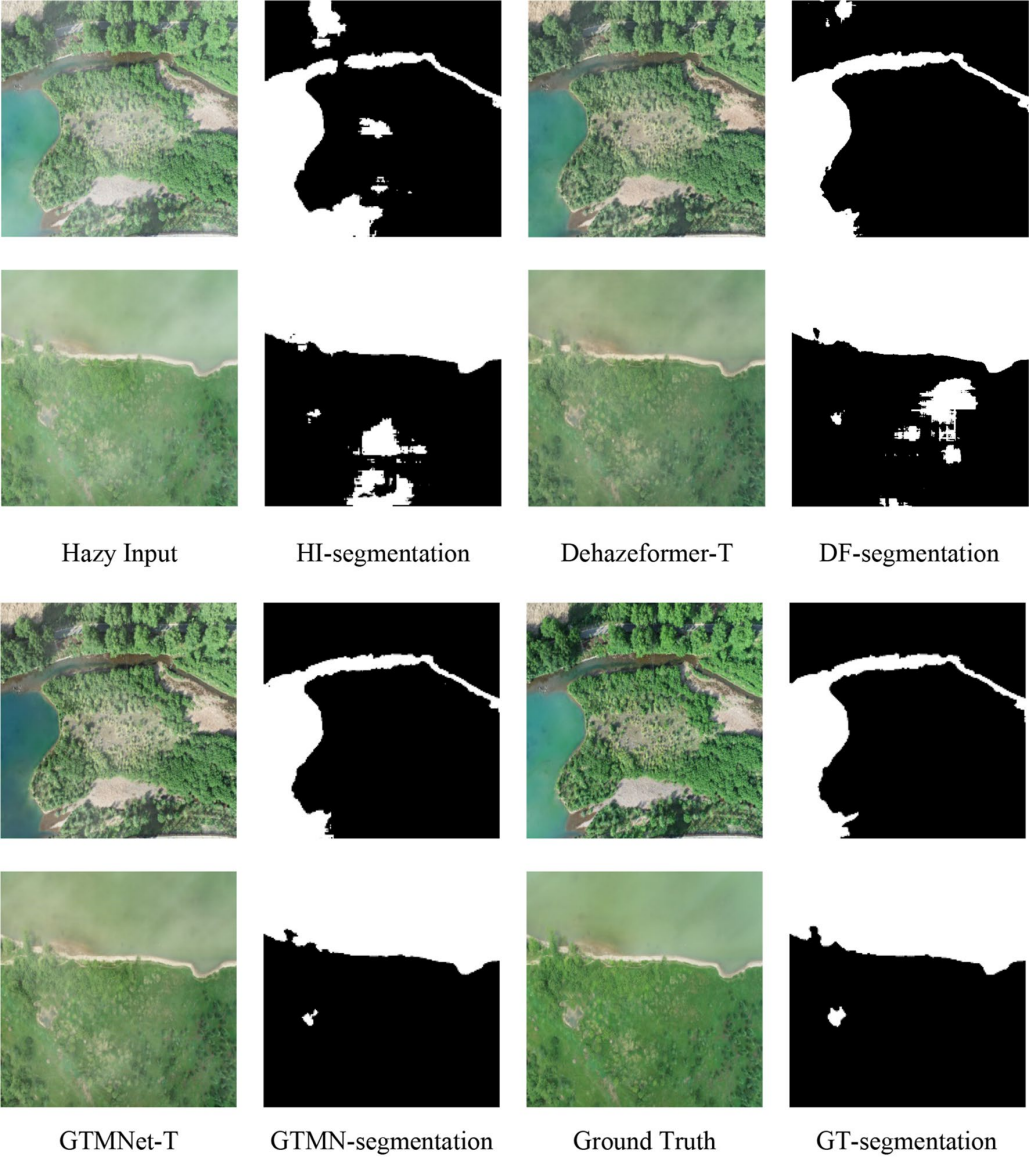
Qualitative comparison of different dehazing results in RSIs water body segmentation task. The ground truths are acquired by a DJI-Phantom 3 Pro.

In this section, we analyze the impact of dehazing results on RSI water body segmentation. Firstly, we trained an RSI water segmentation network inspired by the U-Net for biomedical image segmentation^28^ using 1500 RSIs and tested it using 300 RSIs. Secondly, we selected two images from the test set, added a moderate concentration of haze using Photoshop software, and tested the two images using the -T model trained on Moderate Fog. Finally, we qualitatively compare the results of water body segmentation for hazy inputs, dehazing results from GTMNet-T and Dehazeformer-T, and haze-free images. As shown in Fig. 12, there is very little visual difference between the dehazed images of GTMNet-T and haze-free images. However, the dehazed images of Dehazeformer-T have increased errors in the water body segmentation process compared to haze-free images.

## Conclusions

Combining the advantages of ViT and CNN, we propose a new RSI dehazing hybrid model GTMNet. The GTM is first introduced into the model using two SFT layers to improve the model’s ability to estimate the haze thickness. The SOS boosted module is then introduced to refine the local features of the restored image gradually. The experimental results show that the proposed model has an excellent dehazing effect even for small-scale hazy RSI datasets, compensating for the lack of training data for current low-level visual tasks effectively and improving the model’s applicability. Compared with state-of-the-art methods, GTMNet mitigates, to some extent, color distortion on the roof of buildings with high brightness and in dense haze areas.

We found that the effectiveness of the prior GTM depends on the presence of the SOS boosted module. Therefore, the strategy of introducing external prior knowledge is crucial. In future work, inspired by a dynamic memory network (DMN +)^29^ to fuse target-related external knowledge and image features, and a multi-level features fusion network (MFFN)^30^ to address the network redundancy, we will explore the self-weighted fusion strategy of the auxiliary data (e.g., Synthetic Aperture Radar image, GTM) and RSI features. In addition, we will further study strategies of combining traditional methods and deep learning–based methods, and design more suitable models to avoid overfitting.

## Supplementary Information

Supplementary Information.

## Data Availability

All data generated or analyzed during this study are included in this published article. The version of Photoshop software for creating hazy RSIs is 24.3, which is available at https://www.adobe.com/products/photoshop.html.
