# Supplementary material for: GTMNet: a vision transformer with guided transmission map for single remote sensing image dehazing
Source: Sci Rep. 2023 Jun 7;13:9222. doi: 10.1038/s41598-023-36149-6 (PMC10247807; doi:10.1038/s41598-023-36149-6)
Supplement: Supplementary file 1 — Supplementary Information. [file 41598_2023_36149_MOESM1_ESM.docx]

## Supplementary Material

### More Visual Results for Real-world Images

Figures 1 and 2 present additional visual results of GTMNet on real-world images from Google Earth. We use various symbols to indicate testing configurations. For instance, GTMNet-T-Thin indicates the dehazing results performed solely on the -T model trained on Thin Fog. Moreover, GTMNet-T-Thin-Thick represents the results of performing two rounds of dehazing, i.e., firstly applying the -T model trained on Thin Fog for dehazing, followed by using the -T model trained on Thick Fog for dehazing. Similarly, GTMNet-T-Thin-Moderate designates the results of performing two rounds of dehazing, i.e., firstly applying the -T model trained on Thin Fog for dehazing, followed by using the -T model trained on Moderate Fog for dehazing. The results demonstrate that our method is capable of removing haze from homogeneous hazy RSIs in real-world scenarios, without causing color distortion. However, for non-homogeneous hazy RSIs in real-world scenarios, especially those with dense haze, we have observed that GTMNet-T-Thick-Thin can achieve optimal results.

| 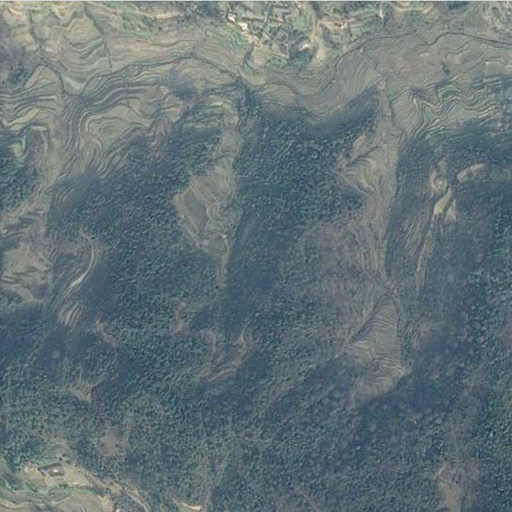 | 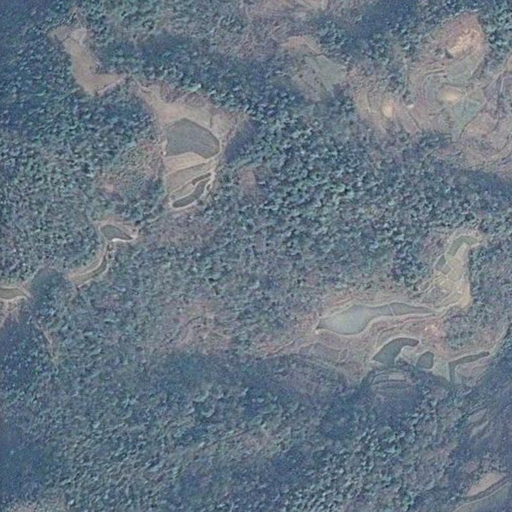 | 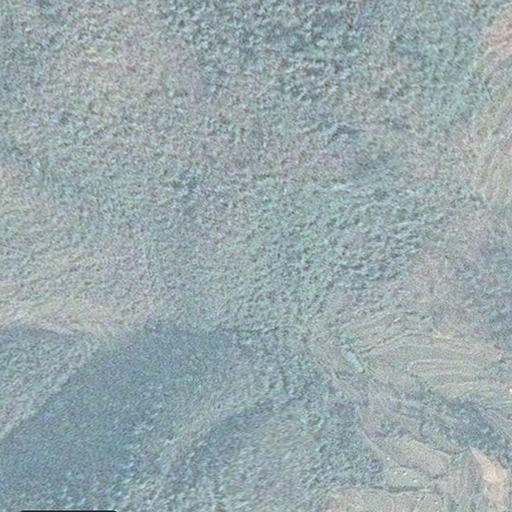 | 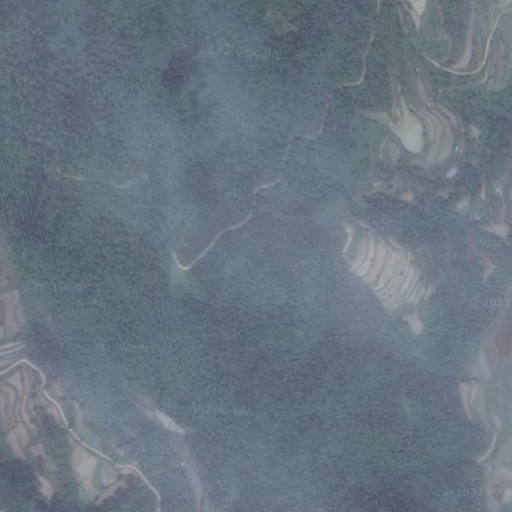 | 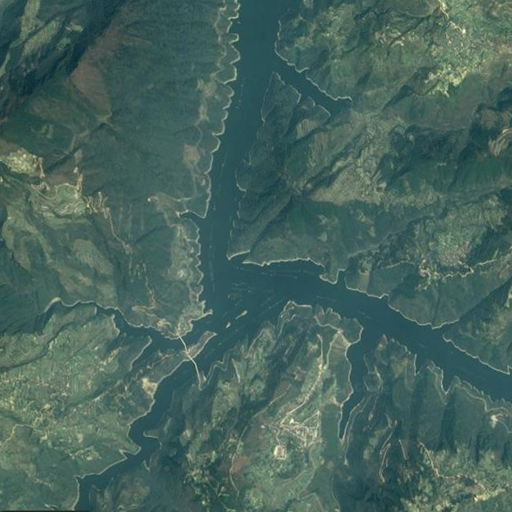 | 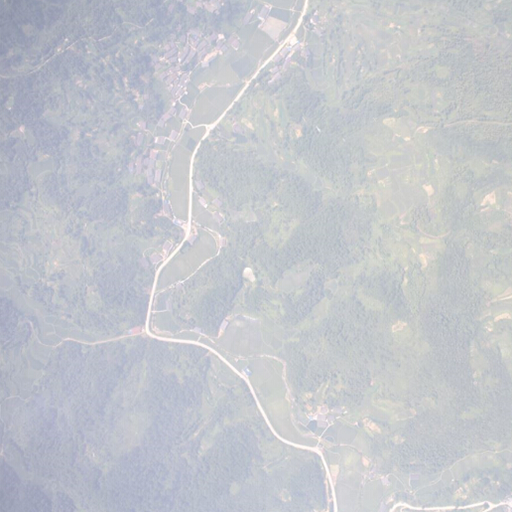 |
| --- | --- | --- | --- | --- | --- |
| Hazy Input | | | | | |
| 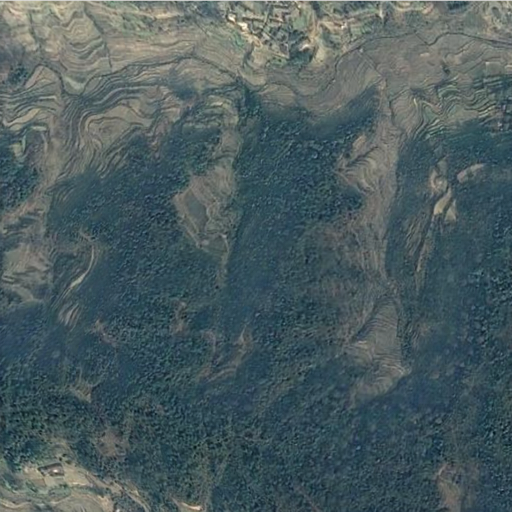 | 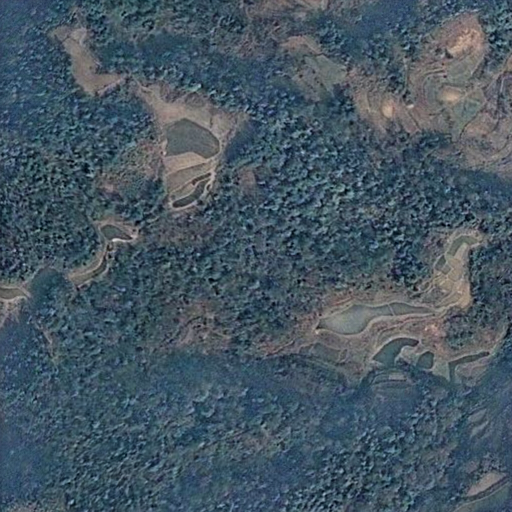 | 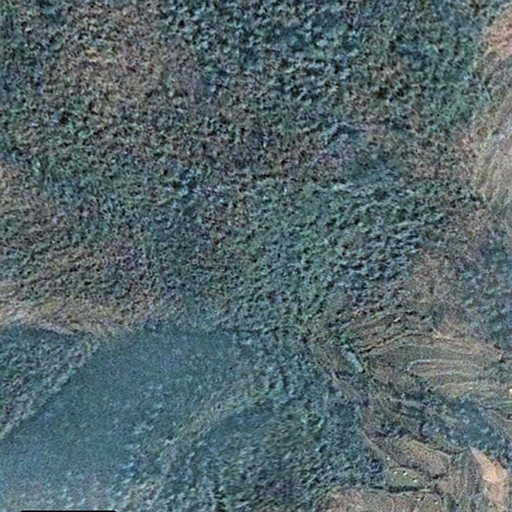 | 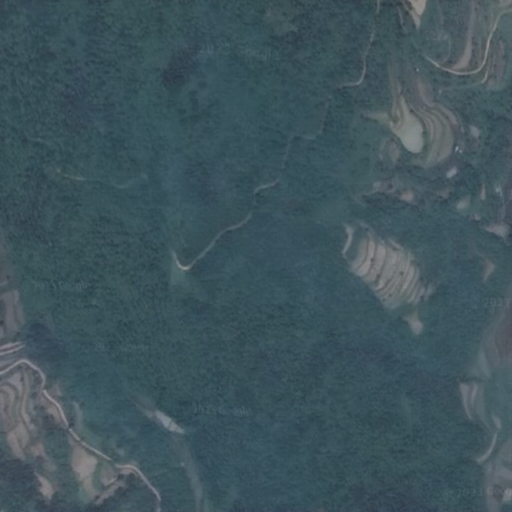 | 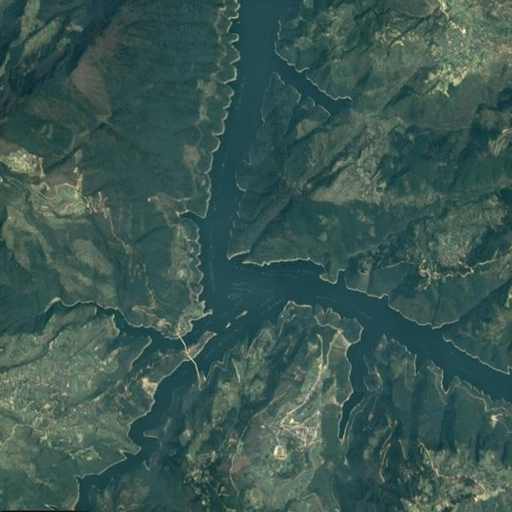 | 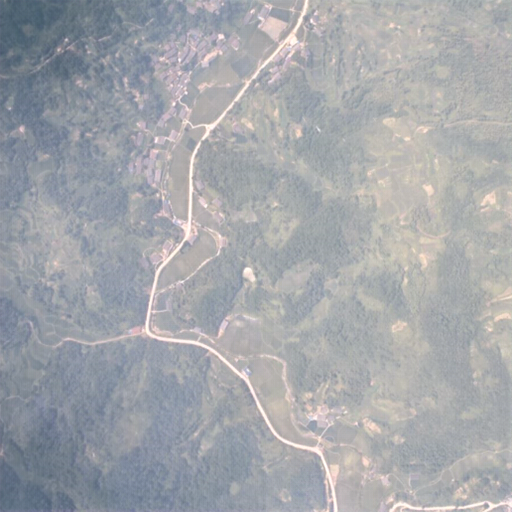 |
| GTMNet-T-Thin | | | | | |
| 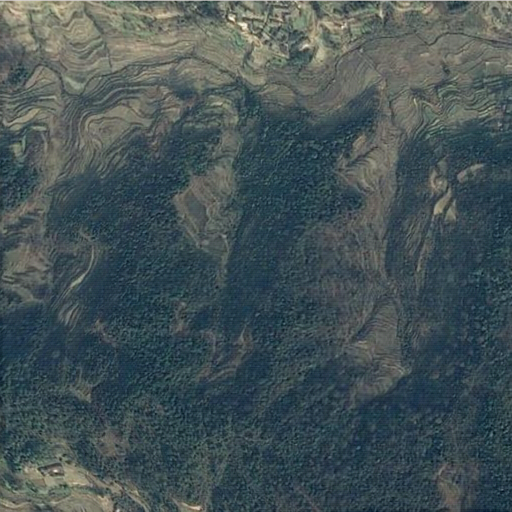 | 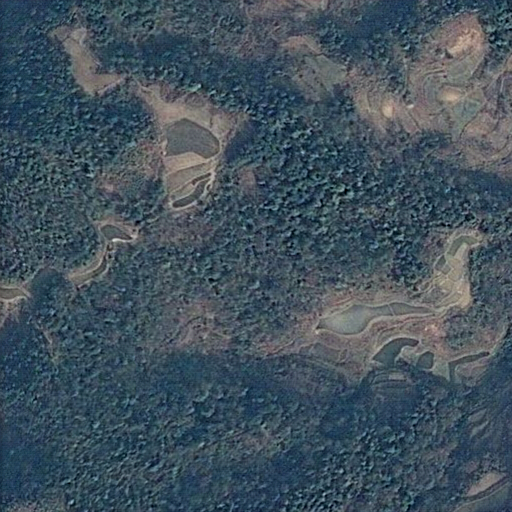 | 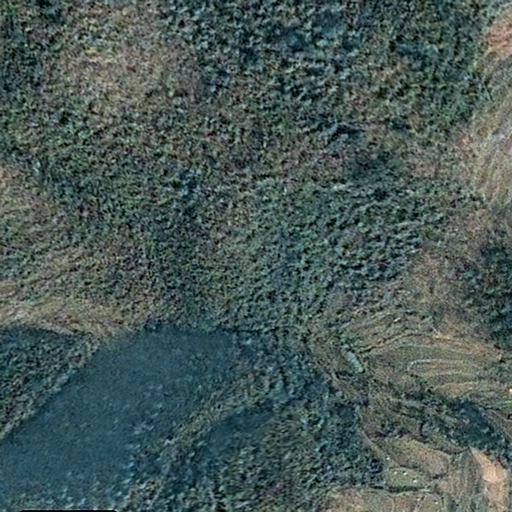 | 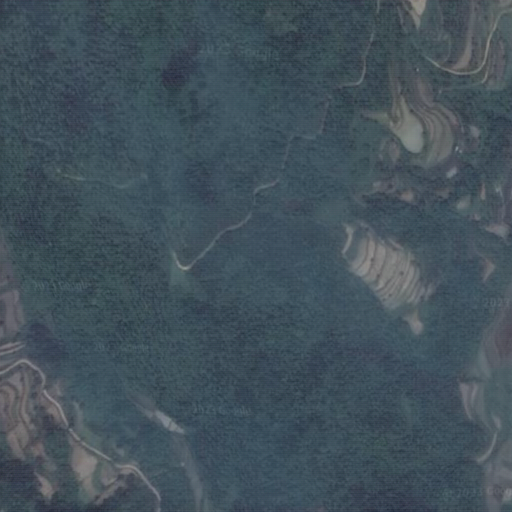 | 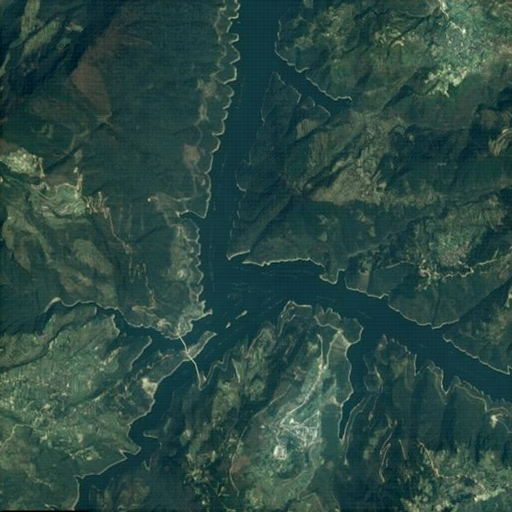 | 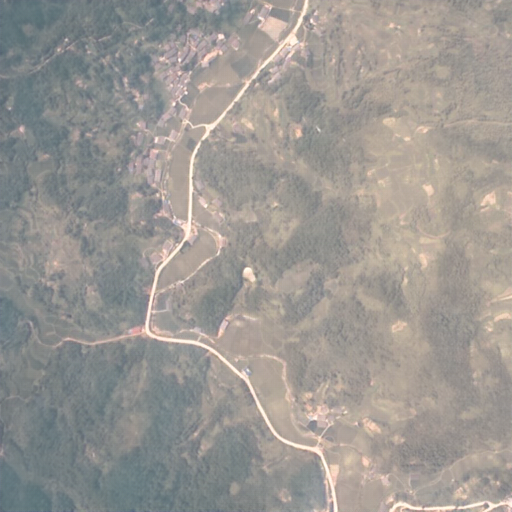 |
| GTMNet-T-Moderate | | | | | |
| 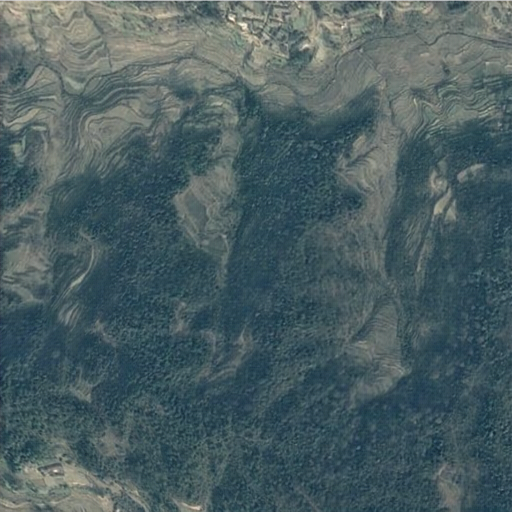 | 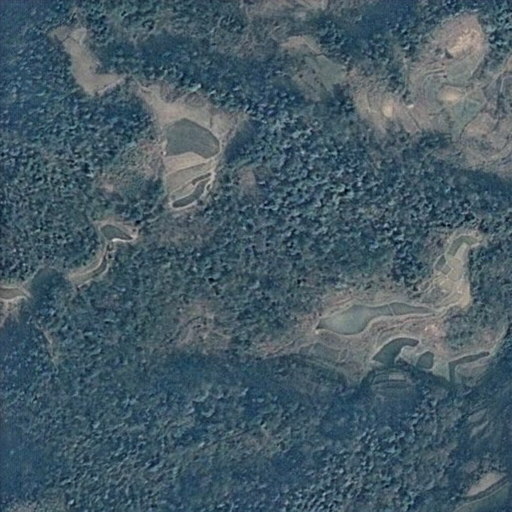 | 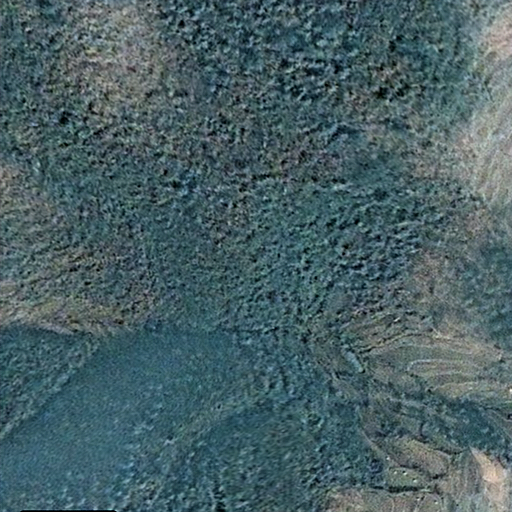 | 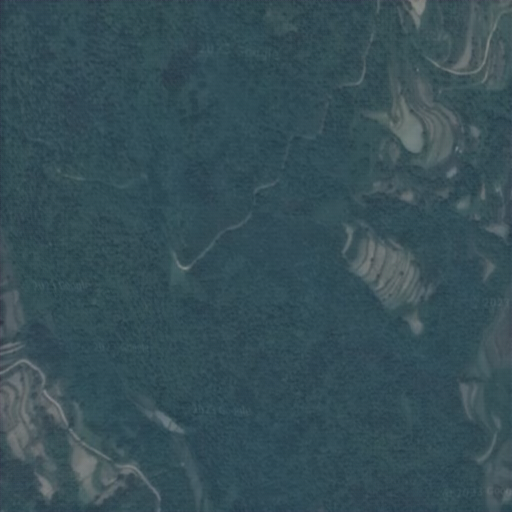 | 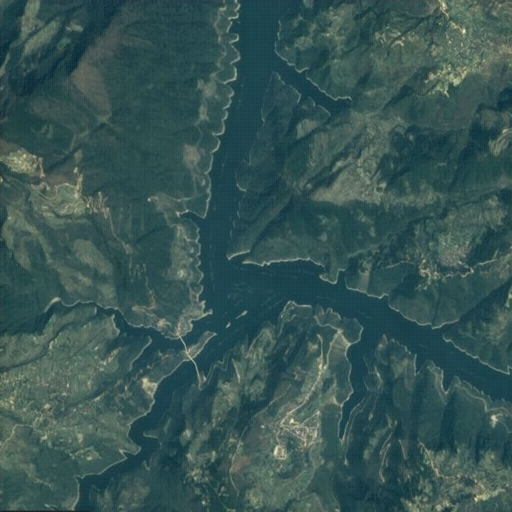 | 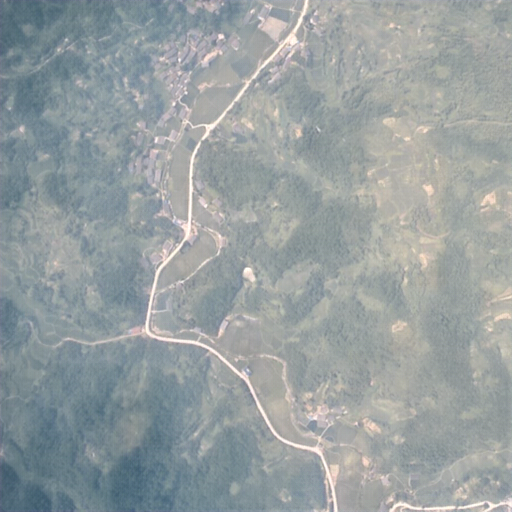 |
| GTMNet-T-Thick | | | | | |

Figure 1. Dehazing results of homogeneous hazy RSIs in real-world scenarios.

| 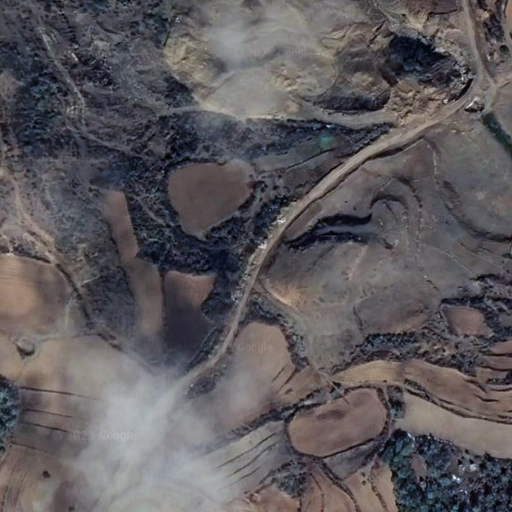 | 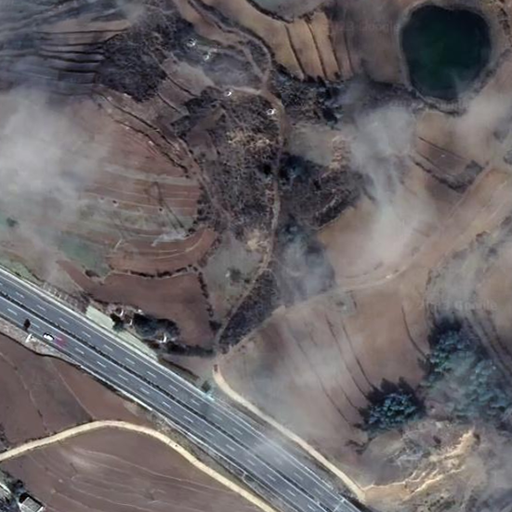 | 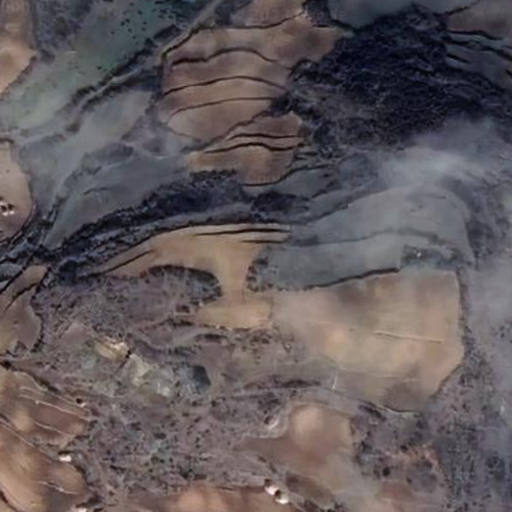 | 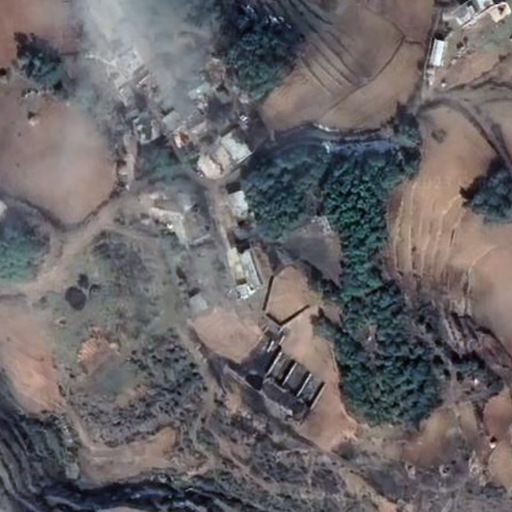 | 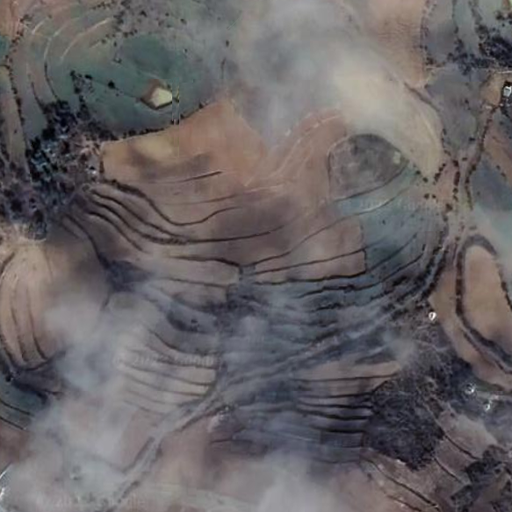 | 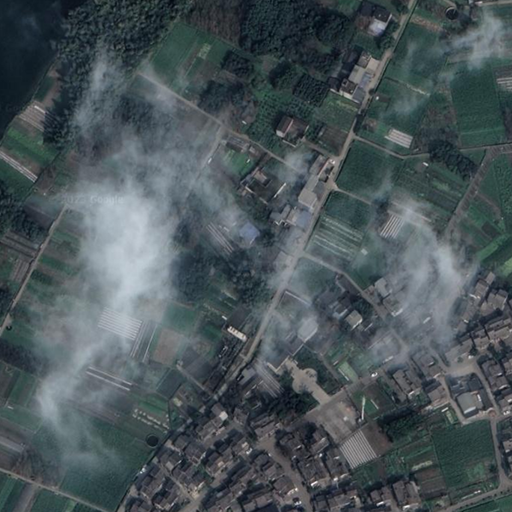 |
| --- | --- | --- | --- | --- | --- |
| Hazy Input | | | | | |
| 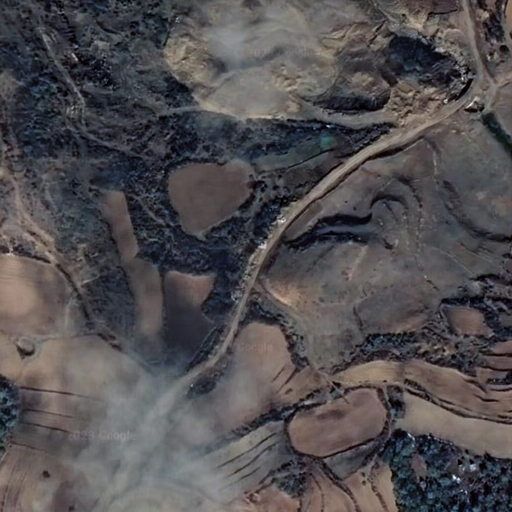 | 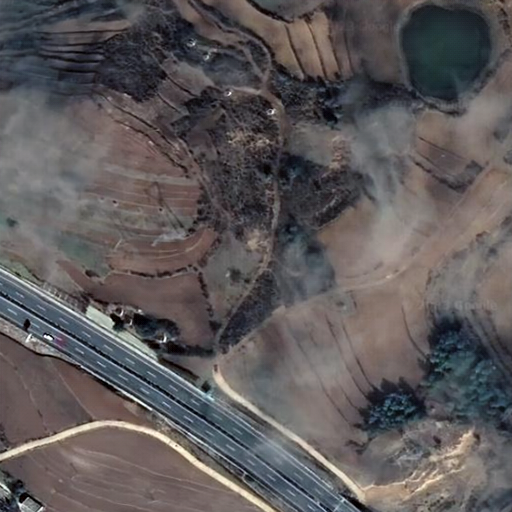 | 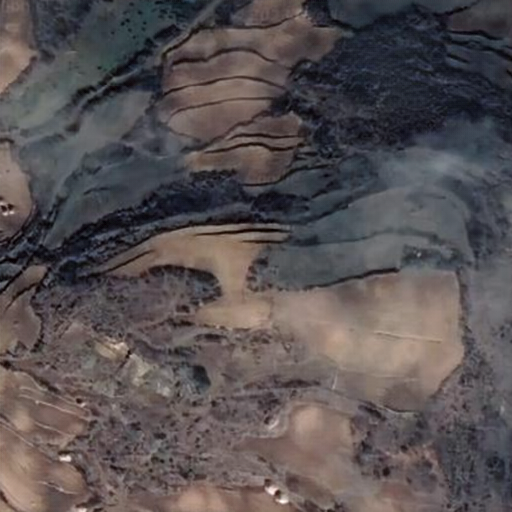 | 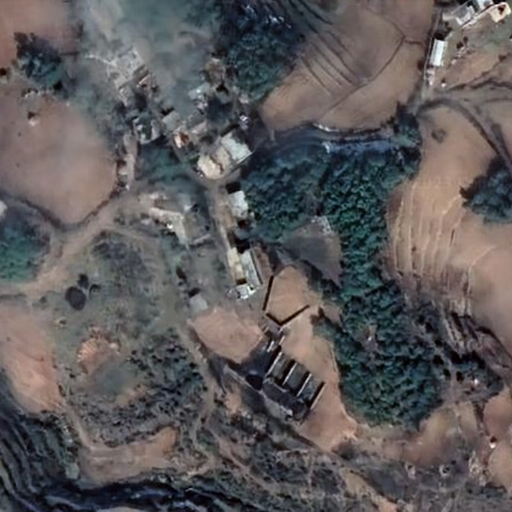 | 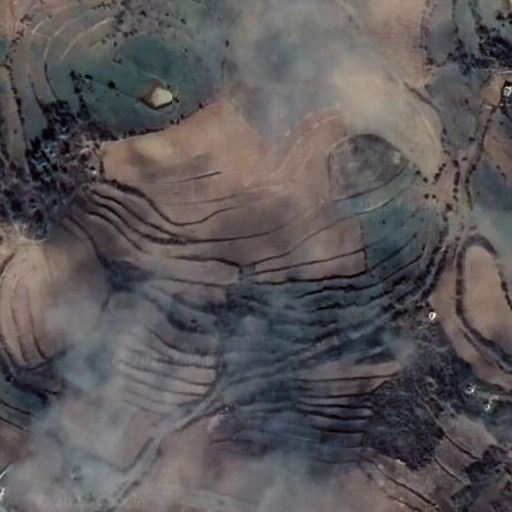 | 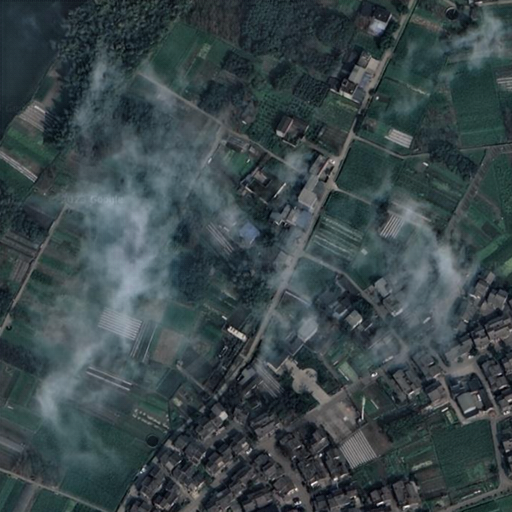 |
| GTMNet-T-Thin | | | | | |
| 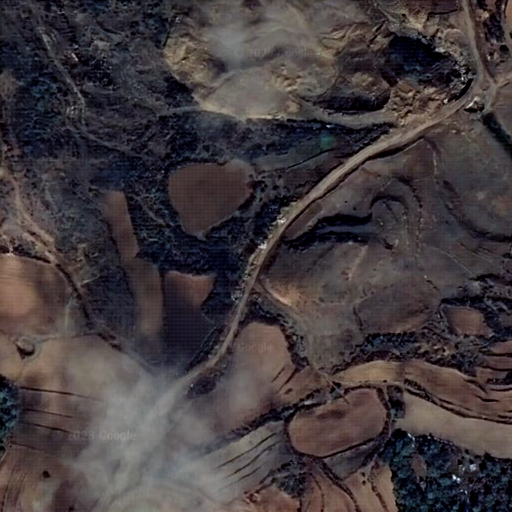 | 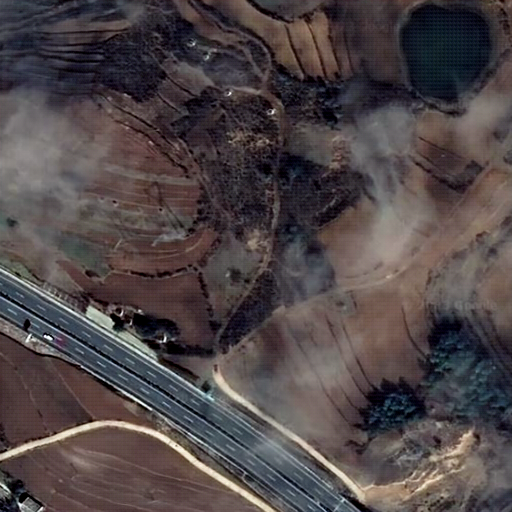 | 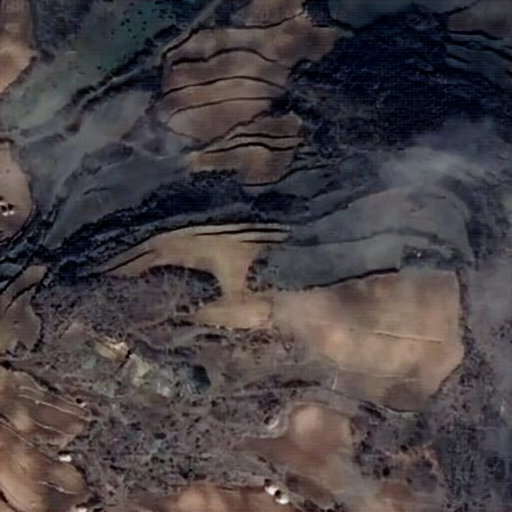 | 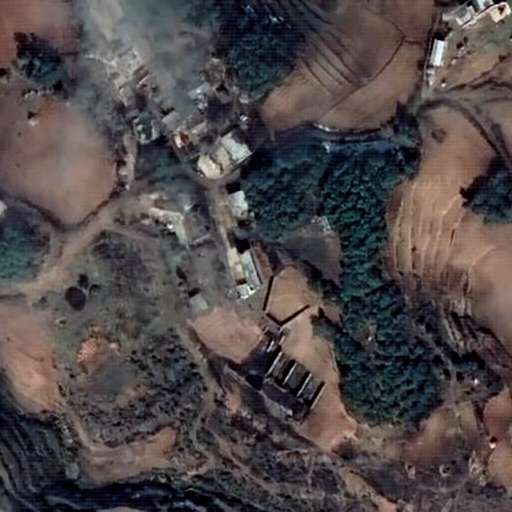 | 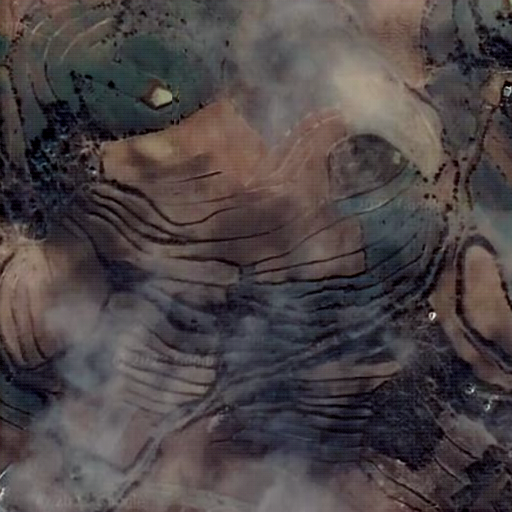 | 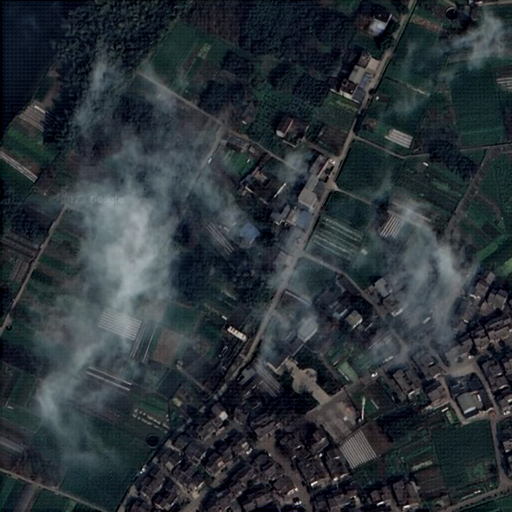 |
| GTMNet-T-Moderate | | | | | |
| 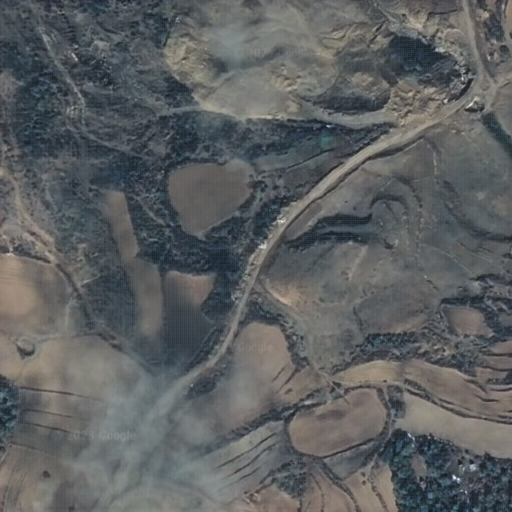 | 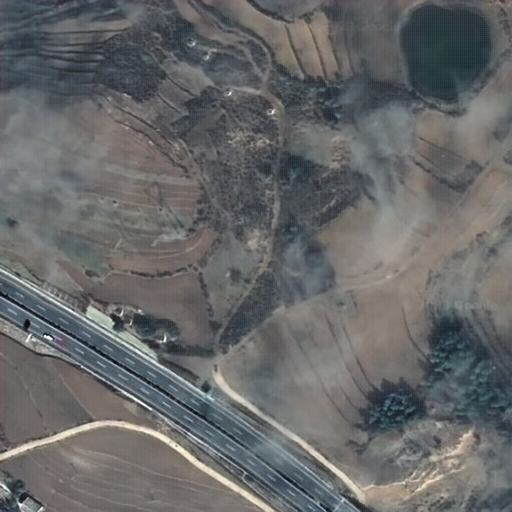 | 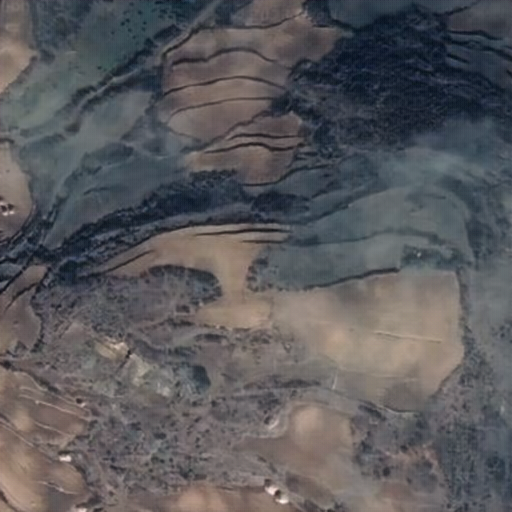 | 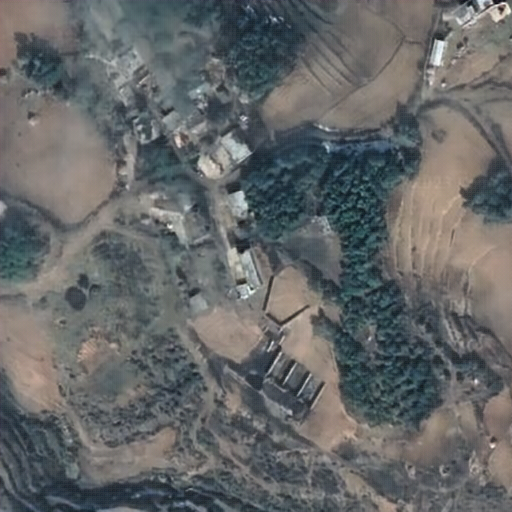 | 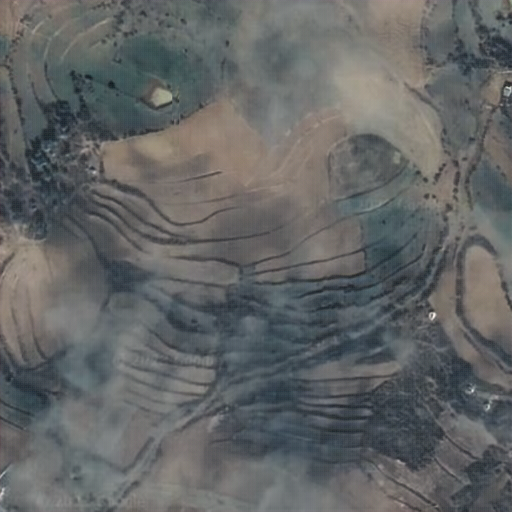 | 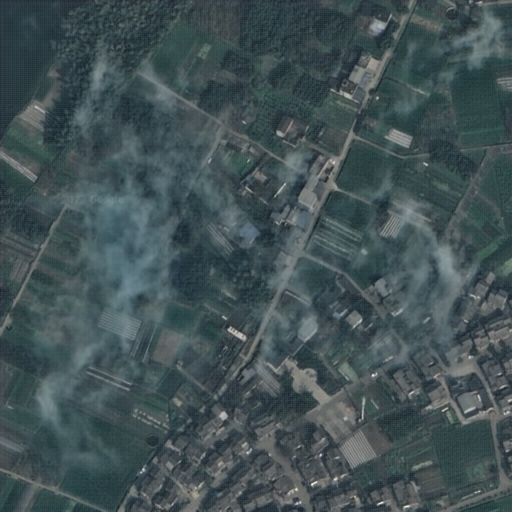 |
| GTMNet-T-Thick | | | | | |
| 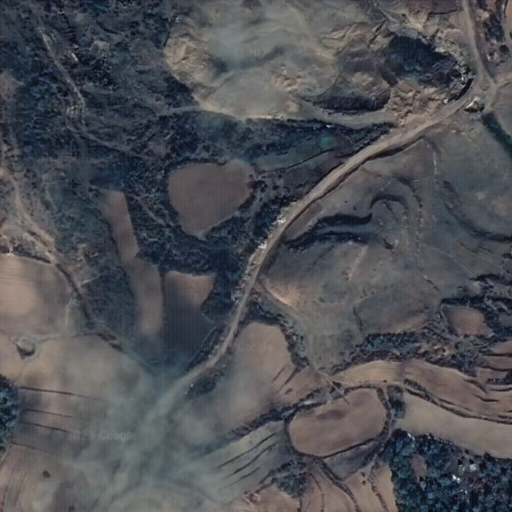 | 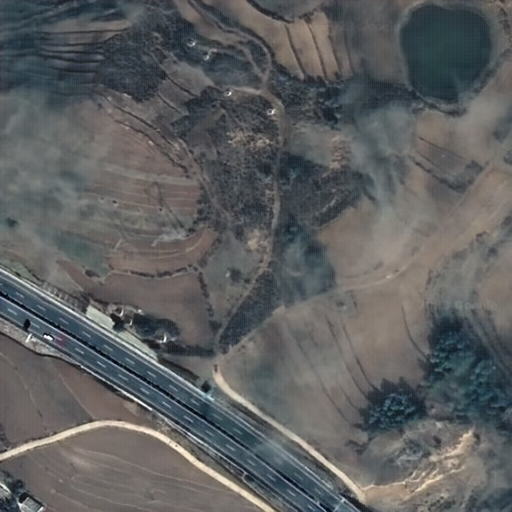 | 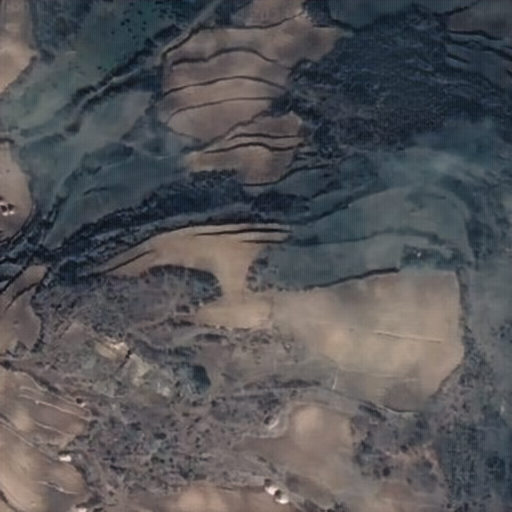 | 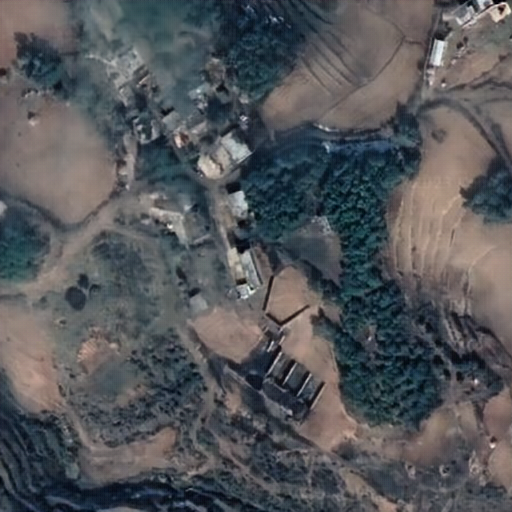 | 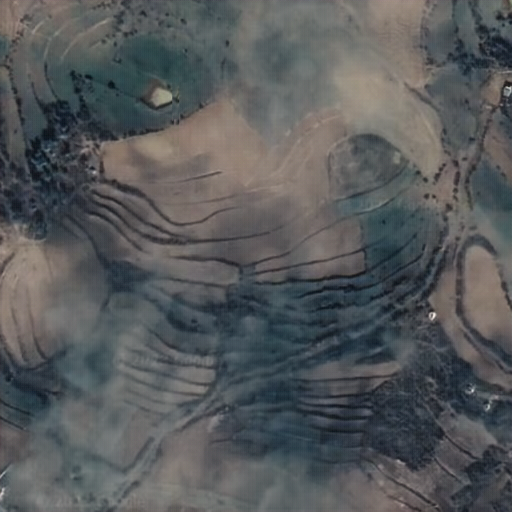 | 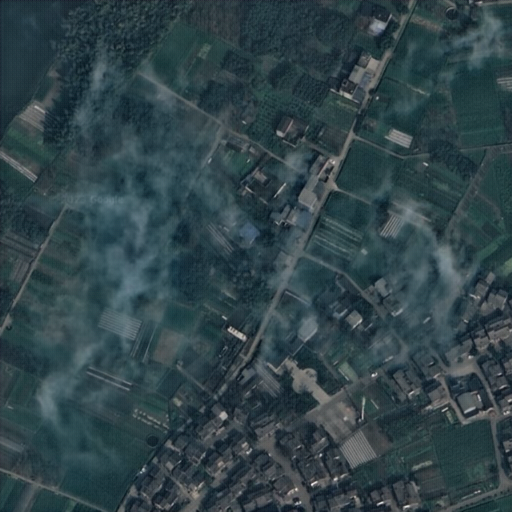 |
| GTMNet-T-Thin-Thick | | | | | |
| 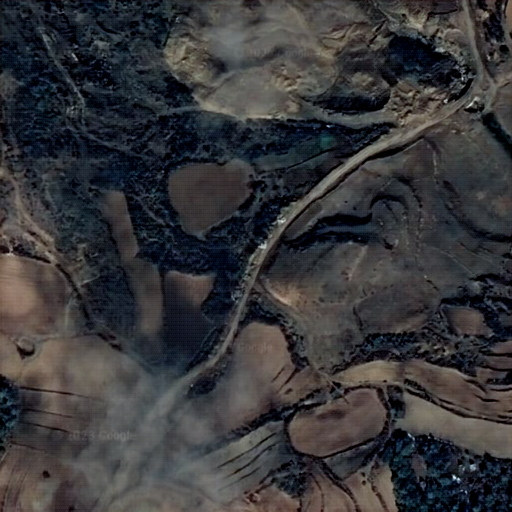 | 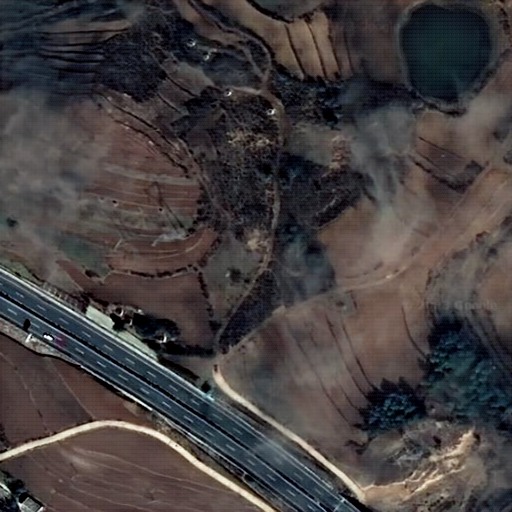 | 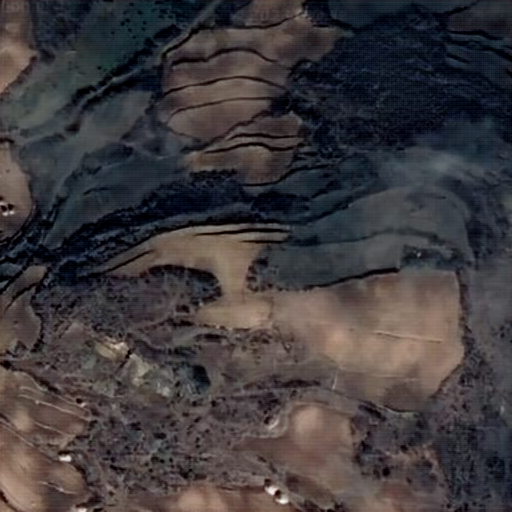 | 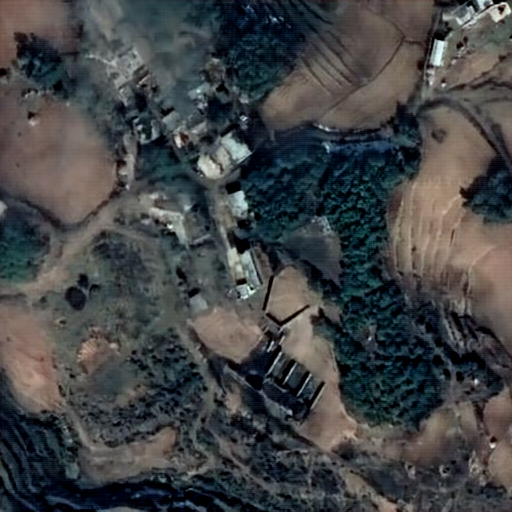 | 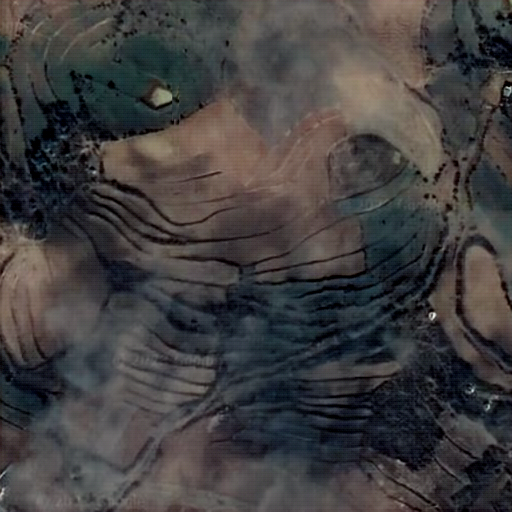 | 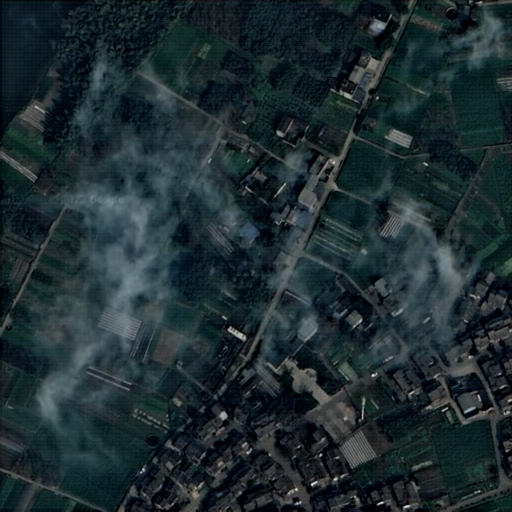 |
| GTMNet-T-Thin-Moderate | | | | | |
| 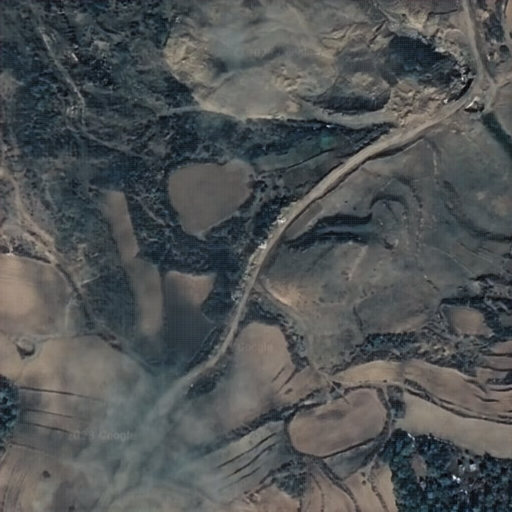 | 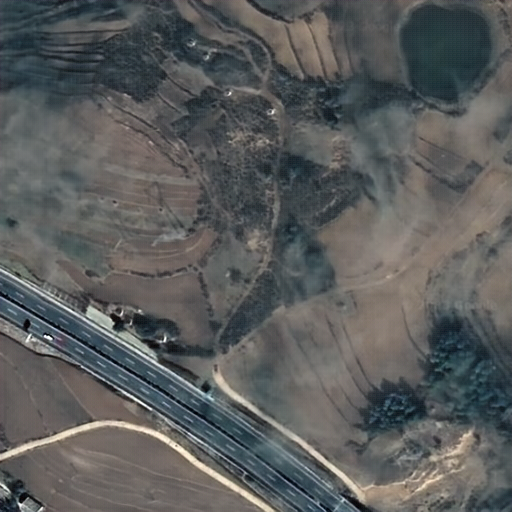 | 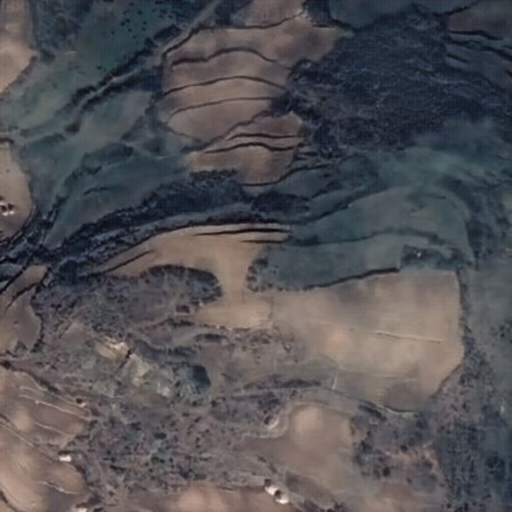 | 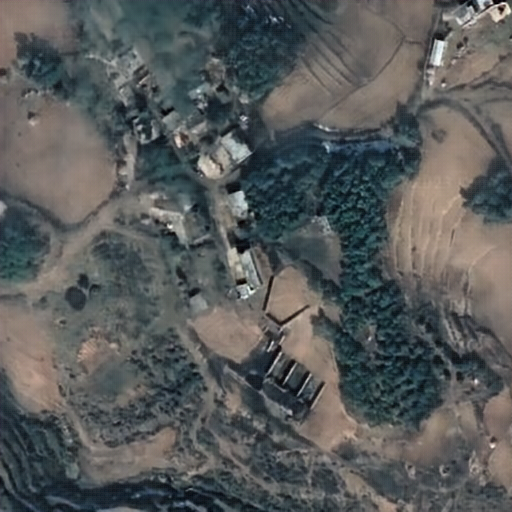 | 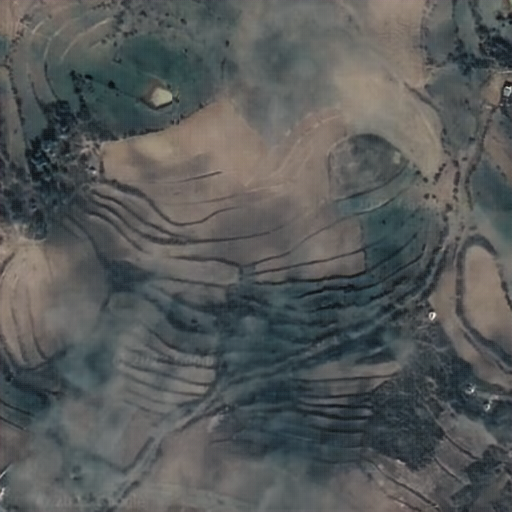 | 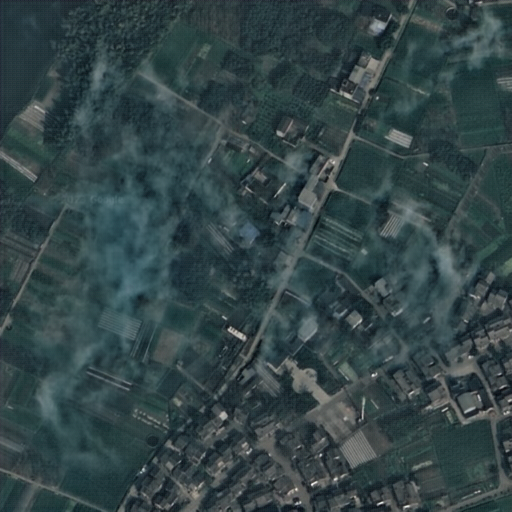 |
| GTMNet-T-Thick-Thin | | | | | |
| 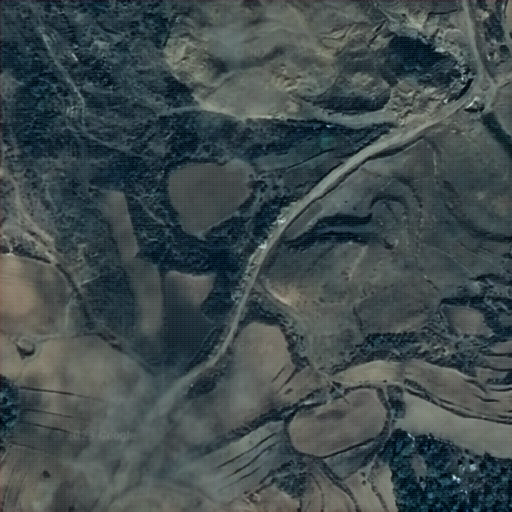 | 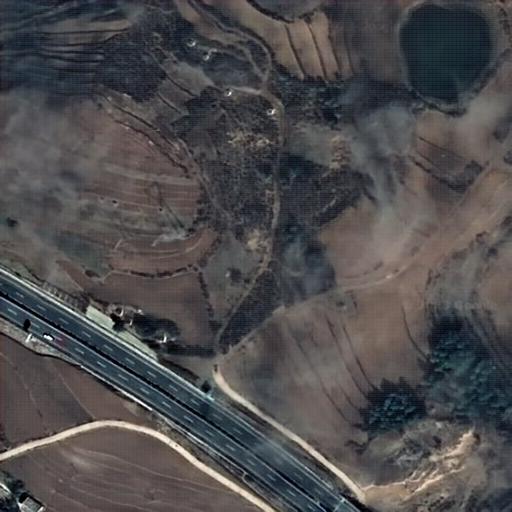 | 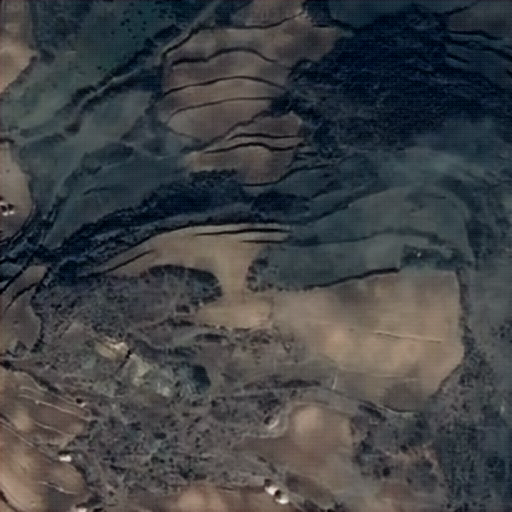 | 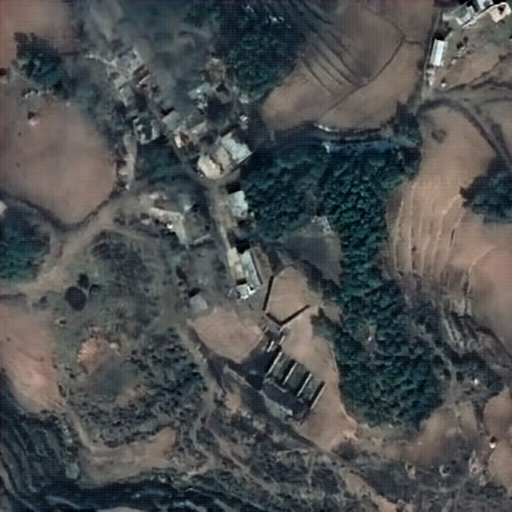 | 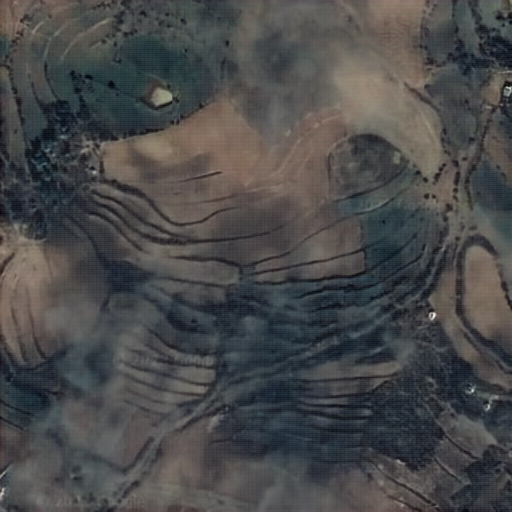 | 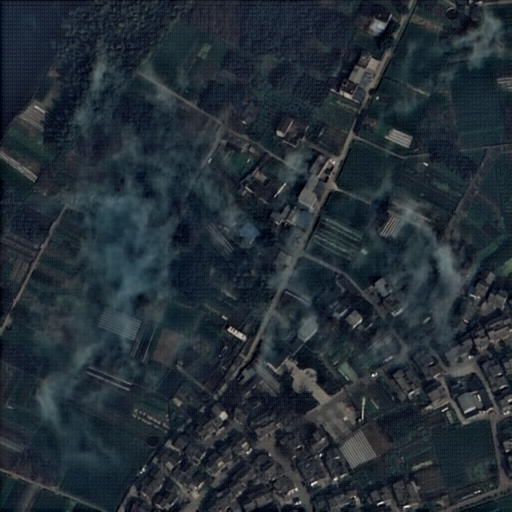 |
| GTMNet-T-Thick-Moderate | | | | | |

Figure 2. Dehazing results of non-homogeneous hazy RSIs in real-world scenarios.
